# Modulation of Global Transcriptional Regulatory Networks as a Strategy for Increasing Kanamycin Resistance of the Translational Elongation Factor-G Mutants in *Escherichia coli*

**DOI:** 10.1534/g3.117.300284

**Published:** 2017-10-18

**Authors:** Aalap Mogre, Reshma T. Veetil, Aswin Sai Narain Seshasayee

**Affiliations:** * National Centre for Biological Sciences, Tata Institute of Fundamental Research, Gandhi Krishi Vigyan Kendra, Bangalore, Karnataka 560065, India; † The Institute of Trans-Disciplinary Health Sciences and Technology, Trans-Disciplinary University, Bangalore, Karnataka 560064, India

**Keywords:** antibiotic resistance, aminoglycosides, kanamycin, transcription factors, gene regulatory networks

## Abstract

Evolve and resequence experiments have provided us a tool to understand bacterial adaptation to antibiotics. In our previous work, we used short-term evolution to isolate mutants resistant to the ribosome targeting antibiotic kanamycin, and reported that *Escherichia coli* develops low cost resistance to kanamycin via different point mutations in the translation Elongation Factor-G (EF-G). Furthermore, we had shown that the resistance of EF-G mutants could be increased by second site mutations in the genes *rpoD*/*cpxA*/*topA*/*cyaA*. Mutations in three of these genes had been discovered in earlier screens for aminoglycoside resistance. In this work, we expand our understanding of these second site mutations, the goal being to understand how these mutations affect the activities of the mutated gene products to confer resistance. We show that the mutation in *cpxA* most likely results in an active Cpx stress response. Further evolution of an EF-G mutant in a higher concentration of kanamycin than what was used in our previous experiments identified the *cpxA* locus as a primary target for a significant increase in resistance. The mutation in *cyaA* results in a loss of catalytic activity and probably results in resistance via altered CRP function. Despite a reduction in cAMP levels, the CyaA^N600Y^ mutant has a transcriptome indicative of increased CRP activity, pointing to an unknown role for CyaA and / or cAMP in gene expression. From the transcriptomes of double and single mutants, we describe the epistasis between the mutation in EF-G and these second site mutations. We show that the large scale transcriptomic changes in the topoisomerase I (FusA^A608E^-TopA^S180L^) mutant likely result from increased negative supercoiling in the cell. Finally, genes with known roles in aminoglycoside resistance were present among the misregulated genes in the mutants.

The efficacy of antibiotics, once heralded as miracle drugs, is now under threat because of the emergence of resistance (Davies 1996; Levy and Marshall 2004). One way in which bacteria become resistant to antibiotics is by gaining genomic mutations. These genomic mutations tend to accumulate mostly in the target of the antibiotic, and often result in a fitness defect because these target genes tend to be essential or important for cell growth (Lenski 1998; Andersson and Levin 1999; Björkman and Andersson 2000; Andersson 2006; Andersson and Hughes 2010). Resistance can also evolve via mutations in nontarget genes (Kern *et al.* 2000), and studying such mutations will yield insight into the mechanism of action of the antibiotic inside the bacterial cell.

Once acquired, resistance can be transferred to sensitive bacteria by horizontal gene transfer (HGT) (Barlow 2009) leading to the concept of a “resistance mobilome” (Martínez *et al.* 2017). The role of bacteriophages, especially superspreaders in HGT is increasingly gaining attention (Keen *et al.* 2017; Lekunberri *et al.* 2017; Touchon *et al.* 2017). Interestingly, bacteriophages could also be used to combat resistance (Merril *et al.* 1996; Lin *et al.* 2017). Other avenues for combating resistance could follow upon the identification of genes necessary for resistance. For example, bacteria can be made hypersensitive to antibiotics by targeting genes involved in resistance with antisense oligomers (Ayhan *et al.* 2016) or the CRISPR-cas system (Goren *et al.* 2017). Once appropriate gene targets are known, these gene-targeting strategies could help prevent emergence of resistance as well as combat resistant bacteria with existing drugs. Crucial to this approach would be understanding the impact of mutations on the activities of the mutated products, *i.e.*, does loss- or gain-of-function result in resistance? Such therapies could complement other strategies *e.g.*, using a combination of antibiotics (Chait *et al.* 2007; Michel *et al.* 2008; Yeh *et al.* 2009; Torella *et al.* 2010; Baym *et al.* 2016).

Aminoglycosides, a group of ribosome targeting antibiotics (Becker and Cooper 2013), have a target that is difficult to modify mutationally. This difficulty arises, especially in fast growing organisms like *Escherichia coli*, because of the presence of multiple copies of the gene encoding the target of aminoglycosides, *i.e.*, the 16S rRNA. On short timescales, it is not possible to mutate all copies of the target gene, seven of which are present in *E. coli* for instance, to achieve resistance (Kotra *et al.* 2000).

In our previous work, we evolved *E. coli* in different sublethal levels of a model aminoglycoside kanamycin (Mogre *et al.* 2014). We obtained multiple kanamycin resistant mutants of the translation Elongation Factor-G (EF-G, encoded by the gene *fusA*). At the lower 4-kan (4 μg/ml; 25% lethal concentration kanamycin), we found a single point mutation in EF-G (FusA^P610T^), whereas, at the higher 8-kan (50% lethal concentration kanamycin), we found two different point mutations in EF-G (FusA^A608E^ and FusA^P610L^). The FusA^P610T^ allele dominated evolved populations for five transfers (rounds of subculture) in 4-kan; whereas, in 8-kan, the FusA^A608E^ allele appeared in the first round of growth, followed by the FusA^P610L^ allele in the next transfer. Among the three EF-G mutants, the FusA^P610L^ allele had the best growth in 8-kan. Interestingly the FusA^A608E^ allele had also accumulated second site mutations in four genes, *viz*., *rpoD*, *cpxA*, *topA*, and *cyaA*, in four different isolates. Apart from our work, evolution experiments in aminoglycosides done by Lázár *et al.* (2013) revealed resistance conferring mutations in *fusA*, *rpoD*, *cpxA*, and *crp* (whose protein product acts downstream of CyaA), but not in *cyaA* and *topA*.

EF-G is a translation factor, and a part of this protein, specifically the tip of domain IV, interacts with the decoding center, the binding site of aminoglycosides (Feldman *et al.* 2010). Thus, whereas the contribution of EF-G—a factor associated with the binding site of the antibiotic—to resistance is easier to understand, the mechanisms by which these second site mutations, in genes not directly related to translation, confer resistance are not immediately apparent. Interestingly, all of the above second site mutations could affect transcription. More specifically, RpoD is the major sigma factor responsible for much of transcription in exponentially-growing *E. coli* (Feklístov *et al.* 2014). CpxA is an envelope stress sensor kinase, which by phosphorylating its response regulator CpxR, activates the expression of genes that tackle membrane stress (Hunke *et al.* 2012). Activation of the Cpx response upon antibiotic exposure was thought to result in increased oxidative stress, and, consequently, cell death (Kohanski *et al.* 2008). However, more recent studies have shown that Cpx activation confers resistance and not sensitivity to certain antibiotics (Mahoney and Silhavy 2013; Manoil 2013). Topoisomerase I (TopA) relaxes negatively supercoiled DNA, and can thereby affect the transcription of many genes. Adenylate cyclase (CyaA) produces cAMP (cyclic adenosine monophosphate), a cellular second messenger, that can influence the expression of a large number of genes via the global regulator CRP (cAMP Receptor Protein) (Zheng *et al.* 2004). Furthermore, Girgis *et al.* (2009) were able to show that disruptions of *cyaA* and *crp* were beneficial to growth in aminoglycosides.

To understand the contribution of these second site mutations to kanamycin resistance, we first generated their single mutant versions. We found that the second site mutations by themselves provide only a marginal increase in growth in kanamycin. These second site mutations, however, allow better growth in kanamycin in either the FusA^P610T^ or the FusA^A608E^ background. By comparing these second site mutants with their corresponding whole gene deletions, we attempted to clarify their roles in kanamycin resistance. Further, using RNA-seq, we found a nonadditive effect between the mutation in EF-G and that in the second-site on gene expression. By comparing our transcriptome data with previously published datasets, along with measurements of plasmid supercoiling, we provide evidence for elevated negative supercoiling in the chromosome of the FusA^A608E^-TopA^S180L^ mutant. Lastly, we were able to see sets of genes with known roles in aminoglycoside resistance among those misregulated in these mutants. This reinforces the idea that mutating promiscuous regulators of transcription might be an effective early strategy for adaptation to stress (Finkel 2006; Wang *et al.* 2010).

## Materials and Methods

### Strain construction

All strains used had the nonpathogenic *E. coli* K12 MG1655 background. RpoD^L261Q^, CpxA^F218Y^, and CyaA^N600Y^ mutants were constructed from their respective FusA^A608E^-RpoD^L261Q^/CpxA^F218Y^/CyaA^N600Y^ double mutants by replacing the FusA^A608E^ allele with the wildtype *fusA* allele linked to a kanamycin resistance cassette. This was done by P1 phage transduction according to the Court laboratory protocol (Thomason *et al.* 2007). Selection with a higher concentration of kanamycin (70–80 μg/ml) ensured that only transductants with the wildtype *fusA* linked to the kanamycin resistance cassette were selected, and that the nontransduced recipient double mutants were not. Background growth of the nontransduced double mutants was a common problem; however, only the best growing colonies were picked since these would contain the kanamycin resistance cassette. All transductants were verified by PCR to ensure the presence of the kanamycin cassette. The kanamycin resistance cassette was flanked by FRT sites, and, thus, was flipped out using the site-specific recombinase Flp provided by the plasmid pCP20. Finally, the temperature-sensitive plasmid pCP20 was cured from these cells by growing them at 42°. The wildtype strain containing the kanamycin cassette near *fusA* was also treated similarly to generate a strain containing the FRT site near *fusA* (WTfrt), and was used as the reference strain. The mutations in *fusA*, *rpoD*, *cpxA*, *topA*, and *cyaA* were checked by Sanger sequencing.

Knockout strains Δ*cyaA*::*kan^R^*, Δ*crp*::*kan^R^* were earlier generated in the laboratory, whereas Δ*cpxA*::*kan^R^* and Δ*cpxR*::*kan^R^* were obtained from Coli Genetic Stock Center (CGSC). These knockouts were transferred into the wildtype used in this study, FusA^P610T^ and FusA^A608E^ strains by phage transduction. The process outlined above, based on the pCP20 plasmid, was used to remove the kanamycin resistance cassette after transferring the gene knockouts to the relevant background strains.

### Growth curves and minimum inhibitory concentration (MIC) determination

Growth curves were performed in Lysogeny Broth (LB) in either flasks or 96-well plates. For the purpose of sample collection and RNA extraction, growth curves were performed in flasks at 37°, 200 rpm with optical density readings measured at 600 nm using a Metertech SP-8001 Spectrophotometer. For strain comparisons, growth curves were performed in 96-well plates. These growth curves were performed using the Tecan Infinite F200pro plate reader. The machine incubated the plate at 37°, and carried out shaking at 198 rpm with optical density readings measured at 600 nm every 15 min.

MICs were measured as previously described, using a modification of the broth dilution technique (Mogre *et al.* 2014).

### Cyclic adenosine monophosphate (cAMP) estimation

Estimation of intracellular cAMP levels was carried out using the cyclic AMP Select EIA kit (501040; Cayman Chemical). Cells growing exponentially (∼1.5 hr in LB) and in the stationary phase (∼12–15 hr in LB) were harvested by centrifugation at 13,000 × *g* for 1 min. Cells were immediately transferred onto ice to prevent breakdown of cAMP by phosphodiesterases. Cell pellets were washed once with TBST (20 mM Tris, 150 mM NaCl, 0.05% Tween 20, pH 7.5) before being resuspended in 0.05 *N* HCl. Cells were then boiled for 5 min to extract cAMP. Cells were then spun down at 14,000 × g and the supernatant containing cAMP was collected. Estimations of cAMP were carried out according to the kit’s instructions with the exception that the provided cAMP standard was diluted in 0.05 *N* HCl to generate the standard curve, since HCl was used for the extraction process.

### Chloroquine gel analysis

Overnight grown cultures of wildtype, FusA^P610T^, FusA^A608E^, and FusA^A608E^-TopA^S180L^, transformed with the pUC18 plasmid, were diluted 1:1000 in 25 ml LB in a 250-ml flask and grown to exponential (∼0.3 OD_600_) and stationary phase (24 hr). pUC18 extraction was done using the QIAprep Spin Miniprep Kit (Qiagen, Valencia, CA). About 250 ng plasmid was loaded in a 0.8% agarose gel containing 2.5 μg/ml chloroquine made in Tris Borate EDTA (TBE) buffer. Samples were run in TBE at 3 V/cm for 17 hr. After the run, the gel was washed with water for 24 hr to remove the chloroquine. After the wash, the gel was stained with 1 μg/ml ethidium bromide solution for 1 hr, and destained with water for 3 hr. The gel was illuminated with UV to visualize the different plasmid topoisomers. At the concentration of chloroquine used in these experiments, more supercoiled forms of the plasmid migrate further in the gel. Two biological replicates were analyzed for each strain and the results of one are shown.

### Evolution in kanamycin

For a description of the evolution experiment resulting in the isolation of mutants used in this study, refer to Mogre *et al.* (2014). The MIC of kanamycin of the FusA^P610T^ mutant was ∼60 μg/ml; 25% of this concentration, *i.e.*, 15 μg/ml, was selected for evolving FusA^P610T^ toward higher resistance. Evolution experiments were carried out by batch transfers in LB with and without kanamycin. Two overnight grown replicate populations of *E. coli* were diluted 1:100 in 100 ml LB containing 15 μg/ml kanamycin and 100 ml plain LB as control. Thus, two populations evolving in 15 μg/ml kanamycin, and two control populations evolving in plain LB were incubated at 37°, 200 rpm for 24 hr before the next transfer. Each evolving population was transferred by 1:100 dilution into fresh medium. The concentration of kanamycin was not changed during the course of the evolution experiment. MICs of all populations were followed at the end of each transfer.

Genomic DNA was extracted from both control and experimental populations using the GenElute Bacterial Genomic DNA Kit (NA2120; Sigma-Aldrich, St. Louis, MO). Integrity of the extracted genomic DNA was checked on agarose gel, and quality and concentration were checked using NanoDrop UV-Vis Spectrophotometer (Thermo Scientific). Library preparation for deep sequencing was carried out using the Truseq Nano DNA Library Preparation Kit (FC-121-4001; Illumina).

Paired end sequencing was carried out using the Illumina HiSeq sequencer (2 × 100 Cycles) at the Next Generation Genomics Facility, Centre for Cellular and Molecular Platforms (C-CAMP). FASTX (http://hannonlab.cshl.edu/fastx_toolkit/) quality filtered reads were trimmed using CUTADAPT version 1.9.dev1 (Martin 2011), to remove adapter sequences. Error tolerance in identifying adapters was set to 20% and trimmed reads with <30 bases were discarded. These trimmed reads, were then mapped to the *E. coli* K12 MG1655 reference genome (NC_000913.3) using BWA mem version 0.7.5a-r405 (Li and Durbin 2010); paired files were input together at this step. SAMTOOLS version 1.3 (Li *et al.* 2009) was then used to generate the pileup file from the sam files generated by BWA. Finally, the list of single nucleotide polymorphisms (SNPs) and indels was compiled from the pileup file using VARSCAN version 2.3.8 (Koboldt *et al.* 2012).

### RNA extraction, sequencing, and analysis

For RNA extraction, cells were grown in LB and harvested at the point of maximal growth rate (Supplemental Material, Figure S1 in File S2) after the addition of stop solution to stabilize cellular RNA and stop transcription. Two biological replicates were harvested for each strain, including the reference strain WTfrt. RNA was extracted using the hot phenol-chloroform method. DNase treated RNA was depleted of ribosomal RNA using the Ambion Microbe Express Kit (AM1905). RNA was checked for quality using Bioanalyzer (Agilent). Checked RNA was used for library preparation and sequencing. RNA quality checks, library preparation, and sequencing were carried out at Genotypic (India). Briefly, 100 ng of qubit quantified RNA was used for library preparation using the NEXTflex Rapid Directional RNA-Seq kit (5138-08 Bioo Scientific). The library was quantified using qubit, and its quality was checked using Agilent Bioanalyzer before proceeding for sequencing on the Illumina NextSeq 500 sequencer.

FASTX (*http://hannonlab.cshl.edu/fastx_toolkit/index.html*) filtered reads were trimmed using CUTADAPT (Martin 2011), and aligned to the *E. coli* reference genome (NC_000913.3) using BWA. SNP and indel calling was done to ensure that the correct mutations were present in the relevant samples (Figure S2 in File S2). The number of reads mapping to each gene was obtained using custom Python scripts. Correlation of raw read counts between replicates were high (>0.9). Even across different strains, the strength of correlation was high (>0.8). We also checked that genes within operons were similarly expressed (Figure S3 in File S2).

Subsequently the R (R Core Team 2017) package EdgeR (Robinson *et al.* 2010) was used to call differentially expressed genes using a *P* value cutoff of 0.001 (using the Benjamini Hochberg method to control the false discovery rate in multiple testing). Genes and their fold-changes can be found in File S1.

Gene ontology (GO) analysis was carried out using the R package topGO (Alexa and Rahnenfuhrer 2016). *E. coli* gene annotations were obtained from Ecocyc (Karp *et al.* 2014 p. 2014) and GO terms were obtained from the GO Consortium (Gene Ontology Consortium 2015). In topGO, the Fisher test was used to assess significance of enriched gene sets and terms with *P* values <0.01 were considered significant.

### Data availability

RNA-seq data can be found on the NCBI Gene Expression Omnibus database (Edgar *et al.* 2002; Barrett *et al.* 2013) with the GEO Series accession number GSE82343 (http://www.ncbi.nlm.nih.gov/geo/query/acc.cgi?acc=GSE82343). RNA-seq data of *E. coli* MG1655 Δ*cyaA* and Δ*crp* strains can found with the accession number GSE104505. Deep-sequencing data of populations in the 15-kan evolution experiment can be found with the accession number SRP076371, and deep-sequencing data of some strains can be found with the accession number SRP087477 from the NCBI Sequence Read Archive. All scripts are available on Github (https://github.com/aswinsainarain/mogre_kan_secondsite), as well as on http://bugbears.ncbs.res.in/mogre_kan_secondsite.

## Results and Discussion

### Second site mutations increase kanamycin resistance of EF-G mutants

**Figure 1:**
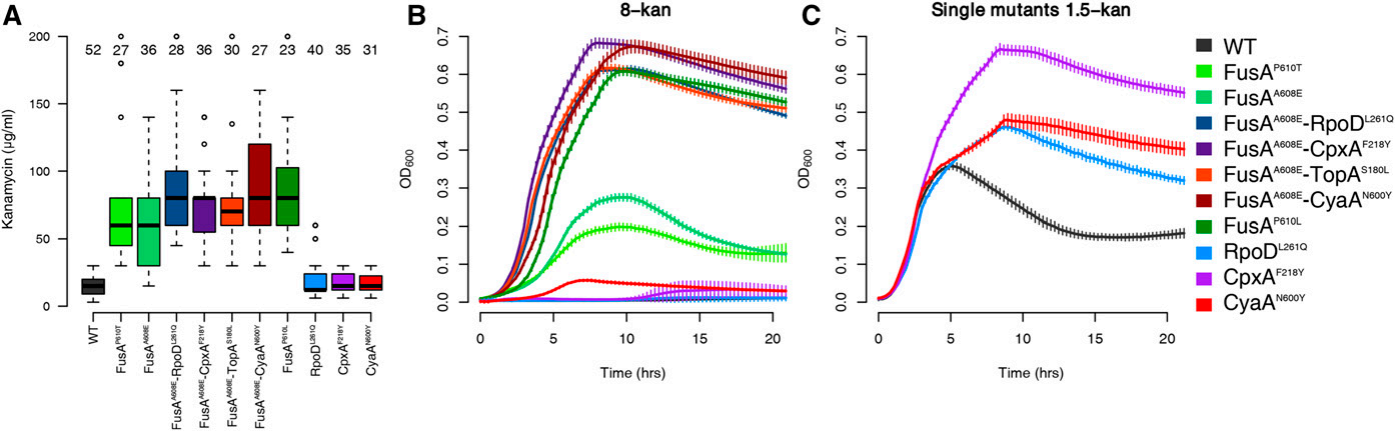
Kanamycin resistance of mutants. (A) Boxplots showing distributions of MICs of kanamycin of the wild type (WT) and various mutants. The number of replicates is mentioned over each boxplot. All mutants, except RpoD^L261Q^, CpxA^F218Y^, and CyaA^N600Y^, are significantly more resistant than the wild type (Welch two sample *t*-test, *P* < 10^−7^). Although the medians of the FusA^A608E^-RpoD^L261Q^/CpxA^F218Y^/TopA^S180L^/CyaA^N600Y^ double mutants tend to be higher than that of the FusA^P610T^ mutant, this difference is not statistically significant (*P* > 0.09). The difference between the medians of these double mutants and the FusA^A608E^ mutant are statistically significant (*P* < 0.02), except in the case of the FusA^A608E^-TopA^S180L^ mutant (P = 0.061) (B) Growth of mutants in 8-kan. (C) Growth of the RpoD^L261Q^, CpxA^F218Y^, and CyaA^N600Y^ mutants in 1.5-kan. In (B) and (C), error bars represent SD of eight replicates. Some part of this data had been generated in our previous work (Mogre *et al.* 2014), with the inclusion of more replicates and data of single mutants.

In our previous work we had found that four “second-site” mutations, namely RpoD^L261Q^, CpxA^F218Y^, CyaA^N600Y^, and TopA^S180L^, appeared on a FusA^A608E^ background in 8-kan. The double mutants had marginally greater resistance to kanamycin, as measured by their MICs, than the single FusA^P610T/A608E^ mutants; this difference was statistically significant in the case of the FusA^A608E^ mutant, but not the FusA^P610T^ mutant (Figure 1A). However, the double mutants grew to a higher cell density in 8-kan, thus pointing to their selective advantage over the single mutant in kanamycin (Figure 1B). Cell sizes of the different mutants were similar, facilitating direct comparisons of optical densities (Figure S4 in File S2). Growth in the presence of kanamycin did not perturb cell size significantly as well (Figure S4 in File S2).

In this work, we replaced the FusA^A608E^ mutation in all the double mutants with the FusA^P610T^ mutation that had evolved in the wildtype background in 4-kan. Previously, we had shown that the FusA^P610T^ mutation showed suboptimal growth under conditions in which FusA^A608E^ and its second-site mutants had emerged (8-kan, Figure 1B). Here, we show that these FusA^P610T^ double mutants grew to a higher cell density than the FusA^P610T^ single mutant alone in 8-kan (Figure S5 in File S2, control growth curves in 0-kan in Figure S6 in File S2). Thus, the selective advantage conferred by these second site mutations was similar between the two primary EF-G mutants, thus further supporting the role of these second site mutations in increasing the resistance of EF-G mutants.

In this work, we constructed single mutant versions of RpoD^L261Q^, CpxA^F218Y^, and CyaA^N600Y^ from their respective EF-G double mutants by replacing the mutant FusA^A608E^ allele with the wildtype allele (see *Materials and Methods*). For reasons not understood, we were unable to construct the TopA^S180L^ single mutant. We saw that the resistance of the double mutants decreased to almost wild-type levels in these single second-site mutants (Figure 1A). This is seen more clearly in the growth curves of these single mutants in 8-kan (Figure 1B). These single mutants, however, fared better than the wild type at a very low concentration of kanamycin (Figure 1C).

From the order of occurrence of mutations in our evolution experiments (Mogre *et al.* 2014), and the inability of single mutants of *cpxA*, *cyaA* and *rpoD* to grow well in kanamycin, it is reasonable to suggest that the mutation in EF-G potentiates the second site mutations, which further increase its resistance.

### Mutation in the extracytoplasmic stress sensor CpxA results in resistance via hyper-activation of the Cpx stress response

The Cpx stress response is mediated by the CpxA sensor kinase and its cognate response regulator CpxR (∼58 gene targets in RegulonDB). This versatile two-component system responds to various kinds of stress signals, especially those associated with membrane stress (Pogliano *et al.* 1997; Raivio and Silhavy 1997; Hunke *et al.* 2012; Vogt and Raivio 2012). Aminoglycosides cause the accumulation of misfolded proteins in the cell membrane and periplasmic space (Bryan and Kwan 1983; Davis 1987; Kohanski *et al.* 2008). The Cpx system responds to this stress (Kohanski *et al.* 2008). It was thought that the crosstalk of the Cpx response with the redox reactive Arc two-component system results in oxidative stress that kills cells (Kohanski *et al.* 2008). However, more recent genetic experiments have revealed that activation of the Cpx response in fact has a protective role (Mahoney and Silhavy 2013).

**Figure 2:**
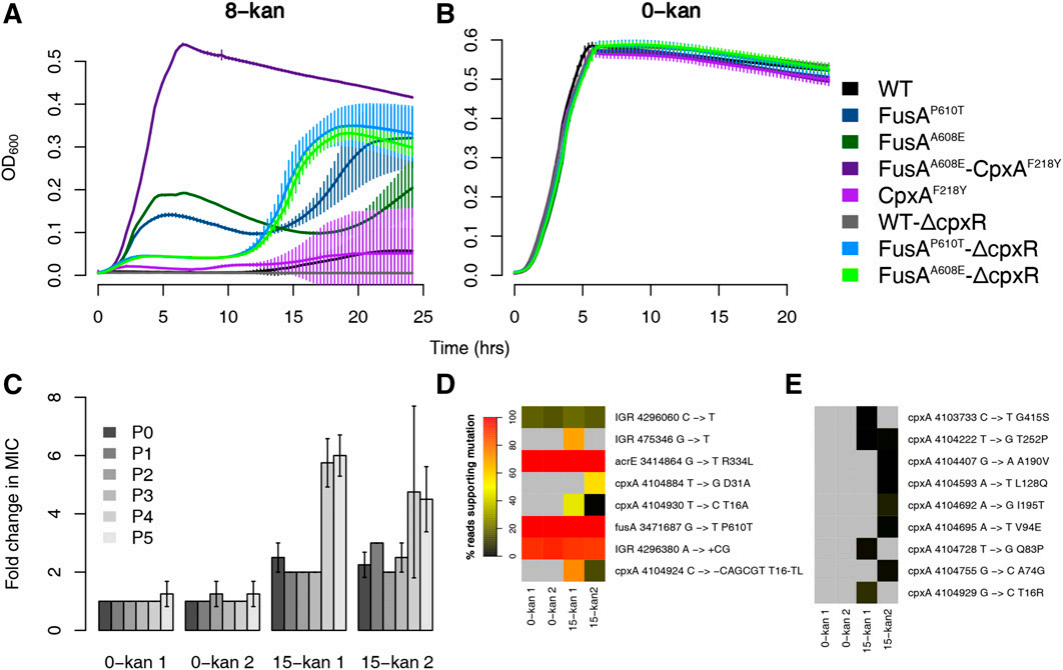
Activation of the Cpx response results in resistance. Growth curves in 8-kan (A) and 0-kan (B). The labels for the *x* and *y* axis are common. Plotted are the means from eight replicates with error bars representing SD. (C) Fold changes in MICs of populations evolved in 15-kan over the MICs of populations evolved in 0-kan. Two replicate populations grown in either 0-kan or 15-kan are shown. MICs of evolving populations at the end of growth (24 hr) after each batch transfer were determined and are represented by P0-P5. The MIC of the 0-kan 1 population at P0 was used to calculate fold changes. Error bars represent SD of four replicates. (D) Heatmaps showing the abundance of variants revealed by sequencing of both control and evolved populations. The color represents the percentage of reads supporting mutations, and is an approximate proxy for the abundance of the mutation. The list of variants was trimmed such that only mutations present in >20% of the reads in at least one sample were retained. (E) Heatmaps showing abundance of all low-frequency *cpxA* variants. The list of variants was trimmed to include only mutations in *cpxA*. The mutations shown in (D) are not shown here. The color scale is as shown in (D).

We sought to understand the effect of the Cpx response on kanamycin resistance, and the impact of the point mutation in CpxA on the Cpx response. For this, we constructed deletion mutants of the response regulator gene *cpxR* in the wild type and in the EF-G mutant background. Deleting the response regulator CpxR, and not the sensor kinase CpxA, is the best way to attenuate the Cpx response. This is because, in the absence of CpxA, CpxR can be cross-activated by other kinases, and this can in fact result in the hyper-activation of the Cpx response (Mahoney and Silhavy 2013). Δ*cpxR* caused a decrease in growth of the background FusA^A608E^ strain in kanamycin. In contrast, Δ*cpxR* had no discernible effect on the wild-type background, which anyhow grows poorly in kanamycin (Figure 2A, *cf*. initial part, *i.e.*, up to 10 hr, of the growth curve). This suggests that an intact Cpx response contributes positively to the resistance of the EF-G mutants.

We saw that the FusA^A608E^-Δ*cpxR* mutants showed a spurt in growth at a certain point in batch culture, whence these mutants grew better than their EF-G single mutant counterparts (Figure 2A). This was consistent across the different transductants tested (data not shown). However, even after this growth spurt, their saturation optical density never reached that of the FusA^A608E^-CpxA^F218Y^ double mutant obtained from the evolution experiment (Figure 2A).

Control growth experiments in the absence of kanamycin show that the decreased growth of the FusA^A608E^-Δ*cpxR* strains in kanamycin, compared to the FusA^A608E^ mutant, was not the result of any generic growth defect conferred by the deletion itself (Figure 2B). Thus, it is reasonable to conclude that the Cpx system is active, and perhaps hyperactive, in the double mutant, and that its activity is linked to resistance.

CpxR has 58 target genes in RegulonDB (Gama-Castro *et al.* 2016). However, only a nonsignificant number of these genes is among the differentially regulated genes in the FusA^A608E^-CpxA^F218Y^ and CpxA^F218Y^ mutants in relation to the wild type (18 in the CpxA^F218Y^mutant and 10 in the FusA^A608E^-CpxA^F218Y^ mutant), as measured by RNA-seq experiments of these mutants in the absence of kanamycin. There are nearly equal numbers of positive and negative targets of CpxR among the upregulated and downregulated genes. Thus, the status of the Cpx response is not clarified by a bird’s eye view of the transcriptome of these mutants. However, *cpxA* and *cpxP* are upregulated in both the FusA^A608E^-CpxA^F218Y^ and CpxA^F218Y^ mutants; and *cpxR* is upregulated in the CpxA^F218Y^ mutant. All these three genes are positive targets of CpxR, and their upregulation in the mutant suggests that the Cpx system might be hyper-active.

### Further evolution of the FusA^P610T^ mutant in kanamycin reveals cpxA as the primary locus targeted for a further increase in resistance

In our previous evolution experiment, while the FusA^A608E^ mutant rapidly accumulated second site mutations in 8-kan, the FusA^P610T^ mutant which had evolved in 4-kan did not accumulate other mutations, even after five transfers (Mogre *et al.* 2014). Thus, we decided to evolve FusA^P610T^ in a higher concentration of kanamycin (15-kan; 15 μg/ml kanamycin; 25% of the MIC of FusA^P610T^) to see what second site mutations would accumulate and eventually dominate in the FusA^P610T^ background. The evolution experiment involved serial batch transfers of pure FusA^P610T^ mutant populations (as defined by a single colony of FusA^P610T^) in 15-kan every 24 hr. MICs of all populations were followed at the end of each transfer throughout the experiment.

We observed an initial increase in MIC of around twofold almost immediately, and it notched up further at the end of the fourth transfer to around fivefold to sixfold (Figure 2C). At the end of the fifth transfer, genomic DNA from both control and evolved populations were sequenced. We looked at the mutations that were present in at least one sample in >20% frequency (Figure 2D). In the populations evolved in 15-kan we saw multiple mutations in *cpxA* present in different frequencies. There were two point mutations and one inframe deletion of six nucleotides in *cpxA*. We also found several other *cpxA* mutations, present at very low frequencies (<20%), only in the populations exposed to kanamycin (Figure 2E). Thus, we see a heterogeneous population with different mutations in *cpxA*. This heterogeneity might be responsible for the large variations in the MIC determinations of these populations (Figure 2C).

Residue changes in CpxA that confer kanamycin resistance were scattered across the protein and were located in helix-I, periplasmic, and cytoplasmic-II domains (Figure S7 in File S2). In particular, mutations in helix-I reached high frequencies (>50%) in the FusA^P610T^ populations evolved in 15-kan. The residue T16 in helix-I was particularly targeted with three different mutations, two of which reached high frequencies. Mutations in the periplasmic domain, helix-II, and cytoplasmic-II domain are known to result in kanamycin resistance due to hyperactivation of CpxA (Raivio and Silhavy 1997). However, we did not see any mutations in helix-II.

We noticed that multiple low frequency mutations in the gene *sbmA* had appeared in the populations evolved in kanamycin (Figure S8 in File S2). The product of this gene is involved in the transport of peptide antibiotics, and its deletion results in increase of resistance to antimicrobial peptides (Laviña *et al.* 1986; Yorgey *et al.* 1994; Salomón and Farías 1995; Saier *et al.* 2009; Corbalan *et al.* 2013; Runti *et al.* 2013; Paulsen *et al.* 2016).

To summarize, the *cpxA* locus seems to be the primary region targeted for the next significant increase in resistance of the EF-G point mutant.

### Disruption of adenylate cyclase catalytic activity gives kanamycin resistance mediated by altered CRP function

Adenylate cyclase is an enzyme that catalyzes the synthesis of cyclic adenosine monophosphate (cAMP) from ATP. cAMP functions as a second messenger in *E. coli* (Botsford and Harman 1992; McDonough and Rodriguez 2011). A well-known mechanism by which cAMP alters gene expression is by binding to and allosterically activating the global transcription regulator cAMP receptor protein (CRP) (Botsford and Harman 1992; McDonough and Rodriguez 2011), which has 477 gene targets in RegulonDB. Girgis *et al.* (2009), subsequent to a transposon mutagenesis screen, demonstrated that deletions of *cyaA* and *crp* increased resistance to aminoglycosides. Furthermore, inactivation of adenylate cyclase was shown to result in activation of the Cpx system (Strozen *et al.* 2005), which has a known role to play in kanamycin resistance (Mahoney and Silhavy 2013).

**Figure 3:**
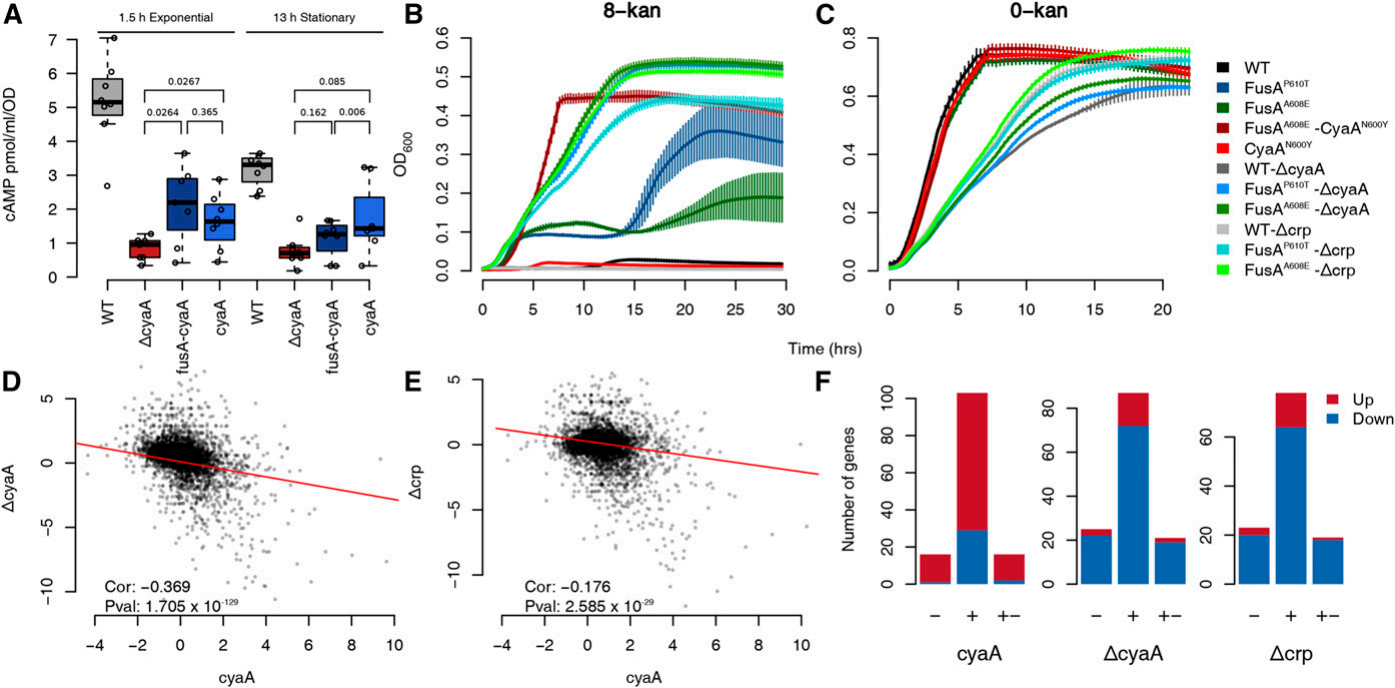
Inactivation of adenylate cyclase results in resistance. (A) Boxplots showing the distribution of estimates of cellular cAMP concentrations of strains in the exponential and stationary phase. The difference between the wildtype and the other mutants are significant (*P* <1  ×  10^−2^). *P* values for other relevant comparisons are mentioned in the plot. The FusA^A608E^-CyaA^N600Y^ mutant is referred to as *fusA-cyaA* and the CyaA^N600Y^ mutant as *cyaA*. (B and C) Growth curves in 8-kan (B) and 0-kan (C). The labels for the *x* and *y* axis are common. Plotted are the means from eight replicates with error bars representing SD. In the 8-kan growth curves, the huge error bars in some of the strains are produced when a few replicates start growing, possibly due to acquisition of some resistance conferring mutation, and thus this error cannot be eliminated. (D and E) Scatter plots comparing log_2_ fold-changes of genes in the CyaA^N600Y^ mutant with those in the Δ*cyaA* (D)/Δ*crp* (E) knockout strains. The mutant is referred to by its gene name for brevity. The time-points for cell harvesting for RNA extraction of the Δ*cyaA*/Δ*crp* strains were similar to that of the mutants. The Spearman correlation coefficient and its *P* value are mentioned. (F) Barplots showing the number of targets of CRP present among the upregulated and downregulated genes in the CyaA^N600Y^, Δ*cyaA* and Δ*crp* strains. The numbers of positive (+), negative (−) and dual targets (+−) of CRP present among the upregulated (red) and downregulated (blue) genes are shown in the stacked barplots. Similar results are seen with the FusA^A608E^-CyaA^N600Y^ mutant and are shown in Figure S9 in File S2.

We found that the levels of cAMP in both the CyaA^N600Y^ and FusA^A608E^-CyaA^N600Y^ mutants were lower than that in the wild type and comparable to that in Δ*cyaA*, thus suggesting loss of function of the mutated adenylate cyclase (Figure 3A). To understand the function of CyaA^N600Y^ and FusA^A608E^-CyaA^N600Y^ further, we deleted *cyaA* and *crp* in the wild-type, FusA^A608E^ and FusA^P610T^ backgrounds. There was an increase in the stationary phase cell density of the FusA^P610T/A608E^-Δ*cyaA* strains in kanamycin compared to the FusA^P610T/A608^ mutants, whereas WT-Δ*cyaA* was not affected considerably, confirming that the loss of adenylate cyclase function results in an increase in kanamycin resistance of the EF-G mutants (Figure 3B). This resistance is possibly mediated via CRP since FusA^P610T/A608E^-Δ*crp* also grew to a higher stationary phase cell density in kanamycin, whereas the WT-Δ*crp* did not (Figure 3B). Both these observations were consistent across multiple transductants tested (data not shown).

Both Δ*cyaA* and Δ*crp*, in the FusA^P610T/A608E^ mutant backgrounds, had lower growth rates in kanamycin compared to FusA^A608E^-CyaA^N600Y^. The presence of a similar growth defect in the wildtype strain containing these gene knockouts, during growth in the absence of kanamycin, indicated that the growth defect was specific to the gene knockouts (Figure 3C), and not the mutants isolated.

We next compared the transcriptomes of the CyaA^N600Y^ mutant with the transcriptomes of the Δ*cyaA* and Δ*crp* strains. Surprisingly, fold changes of genes in CyaA^N600Y^, in comparison to the wildtype, negatively correlated with those in the Δ*cyaA* and Δ*crp* strains (Figure 3, D and E). This correlation was low but significant. This is consistent with the results of our comparison with the list of CRP gene targets available in the RegulonDB database. As expected from the negative correlation, genes that are activated by cAMP-CRP were downregulated in the Δ*cyaA* and Δ*crp* strains (Figure 3F). However, such genes were mostly upregulated in the CyaA^N600Y^ mutant (Figure 3F). This stands for the FusA^A608E^-CyaA^N600Y^ mutant as well (Figure S9 in File S2).

Thus, we show that the CyaA^N600Y^ mutation results in a reduction in catalytic activity. Although the levels of cAMP are low in the CyaA^N600Y^ mutant, their transcriptomes are opposite to that of Δ*cyaA*/Δ*crp*. This is contradictory to expectation, more so since the FusA^P610T/A608E^-Δ*cyaA*/Δ*crp* strains grow better in kanamycin than the FusA^P610T/A608E^ mutants.It is possible that this phenotypic similarity of the FusA^P610T/A608E^-Δ*cyaA*/Δ*crp* strains to the FusA^P610T^-CyaA^N600Y^ mutant is coincidental, and that the full knockout and the point mutant confer resistance through distinct means. We ensured the absence of other mutations in the genomes of the Δ*cyaA*/Δ*crp* transductants using whole genome sequencing (Figure S10 in File S2), and the absence of other mutations in the FusA^A608E^-CyaA^N600Y^ and CyaA^N600Y^ strains by calling mutations from the RNA-seq data. This unexpected behavior of the CyaA^N600Y^ mutation could stem from the fact that the CyaA^N600Y^ mutation is a point mutation, and not a knockout. As a result, the adenylate cyclase protein would still be produced, with residual catalytic activity that can trigger a CRP response. There could also be feedback involved: although not called upregulated, the fold changes of both *cyaA* and *crp* were higher by around twofold in the CyaA^N600Y^ and FusA^A608E^-CyaA^N600Y^ mutants (Figure S11 in File S2). In line with this, the promoter activities of these two genes were also higher in the CyaA^N600Y^ mutant as revealed by promoter-GFP fusions (Figure S11 in File S2). Thus, further work is required to understand the function of CyaA^N600Y^.

Together, we conclude that the evolved point mutation in *cyaA* results in kanamycin resistance via an altered adenylate cyclase and subsequently altered CRP function. This mutation has the added benefit of not conferring the growth defect associated with the knockouts of either of these genes.

### The FusA^A608E^-TopA^S180L^ mutant displays increased negative supercoiling

The bacterial chromosome is a highly condensed and negatively supercoiled DNA molecule (Woldringh 2002; Thanbichler *et al.* 2005; Thanbichler and Shapiro 2006; Toro and Shapiro 2010). The extent of negative supercoiling can influence gene expression (Travers and Muskhelishvili 2005), and is known to be affected by various environmental factors (Rui and Tse-Dinh 2003) such as osmotic stress (McClellan *et al.* 1990; Cheung *et al.* 2003), starvation (Balke and Gralla 1987), temperature (McClellan *et al.* 1990), and oxygen tension (Hsieh *et al.* 1991). Global negative supercoiling of the chromosome is maintained by a balance between the activities of topoisomerases.

**Figure 4:**
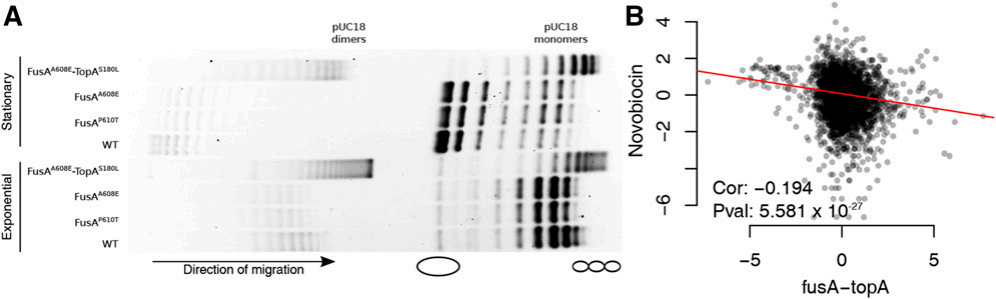
Evidence for supercoiling changes in the FusA^A608E^-TopA^S180L^ mutant. (A) Gel picture showing mobility of pUC18 topoisomers on agarose gel containing 2.5 µg/ml chloroquine. Positions of negatively supercoiled and relaxed forms of the plasmid are indicated by a schematic. (B) Scatter plot showing correlation of log_2_ fold changes of genes in the FusA^A608E^-TopA^S180L^ mutant with microarray derived gene-expression ratios obtained by inhibiting DNA gyrase function using 20 µg/ml novobiocin (data obtained from Peter *et al.* 2004). For a detailed comparison with the Peter *et al.* (2004) dataset, refer to Figure S13 in File S2.

One of the second site mutations in the FusA^A608E^ mutant lies in topoisomerase I (TopA^S180L^), which relaxes negative supercoils. To understand the supercoiling state of the chromosome in the FusA^A608E^-TopA^S180L^ mutant, we employed the cholorquine gel assay (Hsieh *et al.* 1991) to look at the supercoiling of a reporter plasmid: pUC18 (Figure 4A). When plasmid DNA is run through an agarose gel containing 2.5 µg/ml chloroquine by electrophoresis, a separation of different topoisomers is achieved, where more negatively supercoiled topoisomers run further than relaxed topoisomers. In both exponential and stationary phases of growth, we found that the pUC18 molecules were more negatively supercoiled, *i.e.*, they ran further on the chloroquine gel, in the FusA^A608E^-TopA^S180L^ mutant than in the wild type or the single mutants in FusA.

In an earlier gene expression study, Peter *et al.* (2004) had used topoisomerase targeting antibiotics and temperature sensitive mutants in DNA gyrase (a topoisomerase that increases negative superhelicity) to change the supercoiling of the *E. coli* chromosome, and profile the resulting changes in the transcriptome using microarrays. Their data consists of gene expression ratios obtained from microarrays, loaded with RNA from cells at multiple timepoints after treatment/temperature shift, with RNA from cells before treatment/temperature shift serving as a reference. We found that the gene expression profile of FusA^A608E^-TopA^S180L^ mutant negatively correlates with that of novobiocin-treated *E. coli* (Figure 4B). Negative correlation with novobiocin treatment suggests that there is increased negative supercoiling in the cell, since novobiocin inhibits DNA gyrase. This view is supported by the expression levels of the topoisomerases themselves: as expected from a negative feedback in the presence of high negative supercoiling, the levels of *gyrA* and *gyrB* (DNA gyrase) are low and that of *topA* (topoisomerase I) is high (GEO accession number GSE82343). Finally, the FusA^A608E^-TopA^S180L^ mutant shows a slight growth defect at a lower temperature (Figure S12 in File S2), which is in line with the known cold sensitivity of the *topA* deletion strain (Stupina and Wang 2005).

Taken together, we show that the mutation in *topA* reduces its activity and results in increased negative supercoiling in the cell due to intact DNA gyrase function. The loss in activity could also result in increased R-loop formation in the mutant since topoisomerase I resolves R-loops (Massé and Drolet 1999; Usongo *et al.* 2008). Increased translation may help reduce R-loops (Massé and Drolet 1999; Broccoli *et al.* 2004; Gowrishankar and Harinarayanan 2004; Gowrishankar *et al.* 2013). This might even potentially explain the possible genetic interaction between *fusA* and *topA*. Increased dosage of genes encoding topoisomerase IV (*parC* and *parE*) has been shown to relieve growth defects caused by inactivation of *topA* (Kato *et al.* 1990). We also see an increased expression of *parC* and *parE* in the FusA^A608E^-TopA^S180L^ mutant (GEO Series accession number GSE82343).

To our knowledge, we are the first to explore the link between chromosomal negative supercoiling and aminoglycoside resistance. Since we do not have a TopA^S180L^ single mutant, we do not understand the effects of the FusA^A608E^ mutation on the TopA^S180L^ mutation. Further experiments will help work out the biochemical activity of the TopA^S180L^ mutant, its genetic interaction with FusA^A608E^, and the mechanism of resistance of this mutant.

### Similarities among mutants

**Figure 5:**
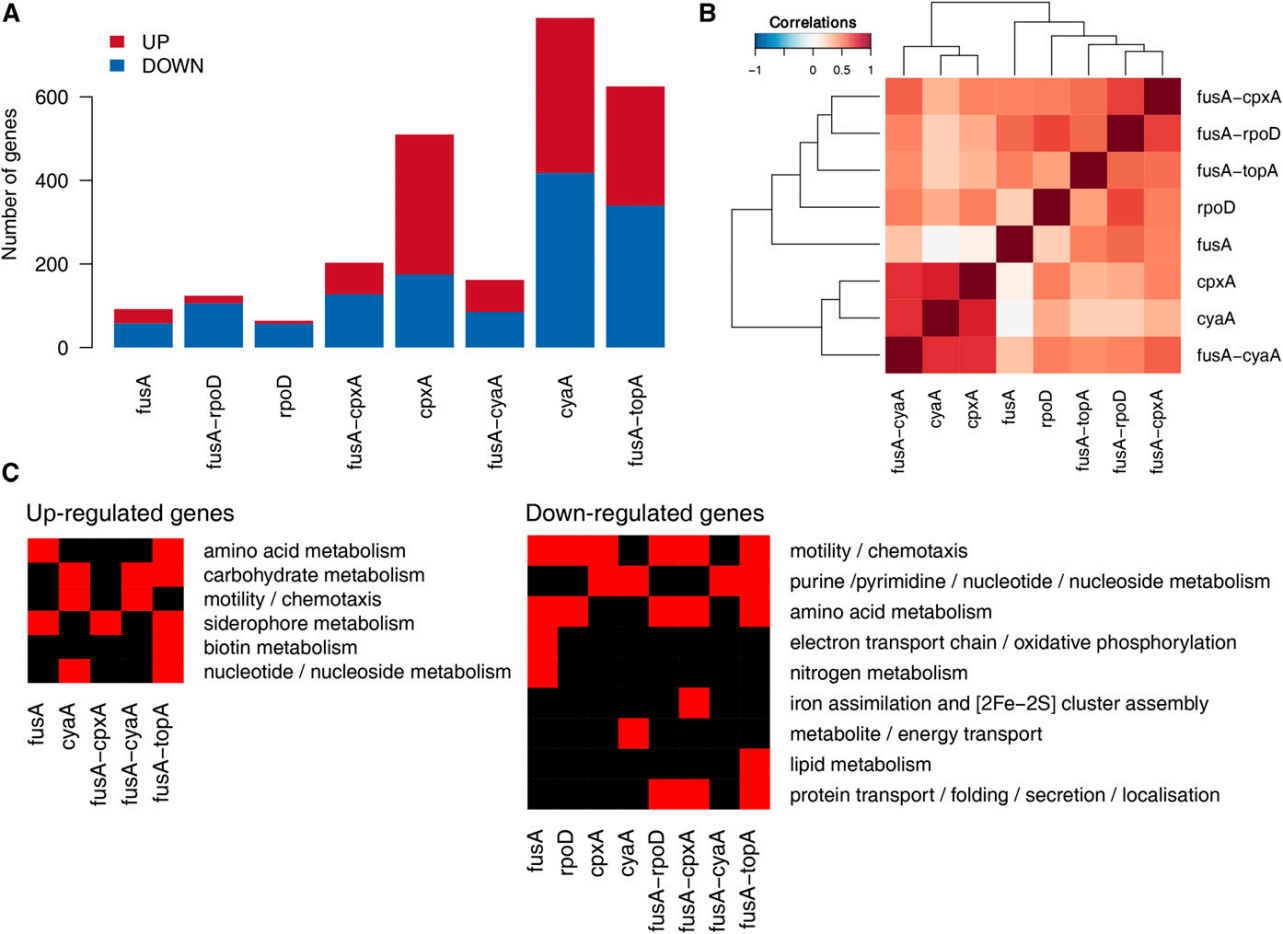
Summary of differentially expressed genes across mutants. (A) Numbers of upregulated and downregulated genes in the mutants. Mutants are referred to by their gene names for brevity. (B) Heatmap showing the matrix of Spearman correlations among mutants. Fold changes of all genes were used to derive these correlations. (C) Heatmap showing enriched GO terms among the upregulated and downregulated genes in the mutants. Many GO terms have been combined to give this simplified picture.

The number of differentially expressed genes varied substantially among the mutants (Figure 5A). The CpxA^F218Y^, CyaA^N600Y^, and FusA^A608E^-TopA^S180L^ mutants had the most number of differentially expressed genes. The FusA^A608E^, RpoD^L261Q^, and FusA^A608E^-RpoD^L261Q^ mutants had the least number of differentially expressed genes.

We were surprised that the *rpoD* mutants had the least number of differentially expressed genes, considering the role of this gene as a transcription initiation factor for ∼50% of the genes in *E. coli*, particularly those expressed in exponentially growing cells. How this mutation results in resistance remains elusive. Nonetheless, the location of the mutation is interesting. The mutation resides in a highly conserved residue in the large, nonconserved, domain of this protein (Figure S14 in File S2). While no clear function is ascribed to this domain, certain residues in this domain have been shown to be involved in promoter escape (Leibman and Hochschild 2007), and one of these residues lies very close to the mutated residue mentioned in our study. It is difficult to hypothesize how a residue involved in allowing the escape of the RNA polymerase from the housekeeping sigma factor to facilitate transcription initiation could be involved in aminoglycoside resistance (unless this is related to coupled transcription and translation), but, in the absence of other information, this remains a valuable lead.

Clustering based on correlation between fold changes of all genes relative to the wild type across mutants tends to cluster the dataset according to the mutants, but not always so (Figure 5B). Notably, fold changes of differentially expressed genes in the CyaA^N600Y^ mutant are well correlated with that in the CpxA^F218Y^ mutant. This further highlights the link between CRP and the Cpx response mentioned earlier (Strozen *et al.* 2005). Thus, as an outcome of the evolution experiment, we see two different mutations resulting in similar transcriptional states. The correlation between the FusA^A608E^-CpxA^F218Y^, FusA^A608E^-RpoD^L261Q^ and FusA^A608E^-TopA^S180L^ mutants were high, offering another example of converging effects of different mutations.

### Effect of second site mutants on gene expression and dependence on EF-G

We evaluated the impact of the FusA^A608E^ mutation on the transcriptomes of the double mutants in detail. The FusA^A608E^ single mutant had roughly 30 genes upregulated, and 50 genes downregulated (Figure 5A). Sequencing of the transcriptomes of single and double mutants enabled us to look at genetic interactions between FusA^A608E^ and the second site mutations, as reflected in the fold changes of differentially expressed genes.

To assess the extent to which the transcriptomes of the FusA^A608E^ single mutant and CyaA^N600Y^/CpxA^F218Y^/RpoD^L261Q^ single mutants explain the gene expression state of the FusA^A608E^-CyaA^N600Y^/CpxA^F218Y^/RpoD^L261Q^ double mutants, we plotted the log_2_ fold change in the double mutant against the sum of the log_2_ fold changes in the FusA^A608E^ single mutant and the second site single mutants (Figure S15, D, H, and L in File S2). In the absence of a genetic interaction between the two single mutants, we would expect the scatter plot to lie along the 45° line. We find that this is not the case in each of the three double mutants we evaluated. In other words, the absolute difference in log_2_ fold change in expression between the double mutant and the sum of the two corresponding single mutants is significantly different from zero (Wilcoxon signed rank test P value <10^−10^). These indicate that the FusA^A608E^ background affects the transcriptional state of the CyaA^N600Y^/CpxA^F218Y^/RpoD^L261Q^ single mutants in a nonadditive manner.

**Figure 6:**
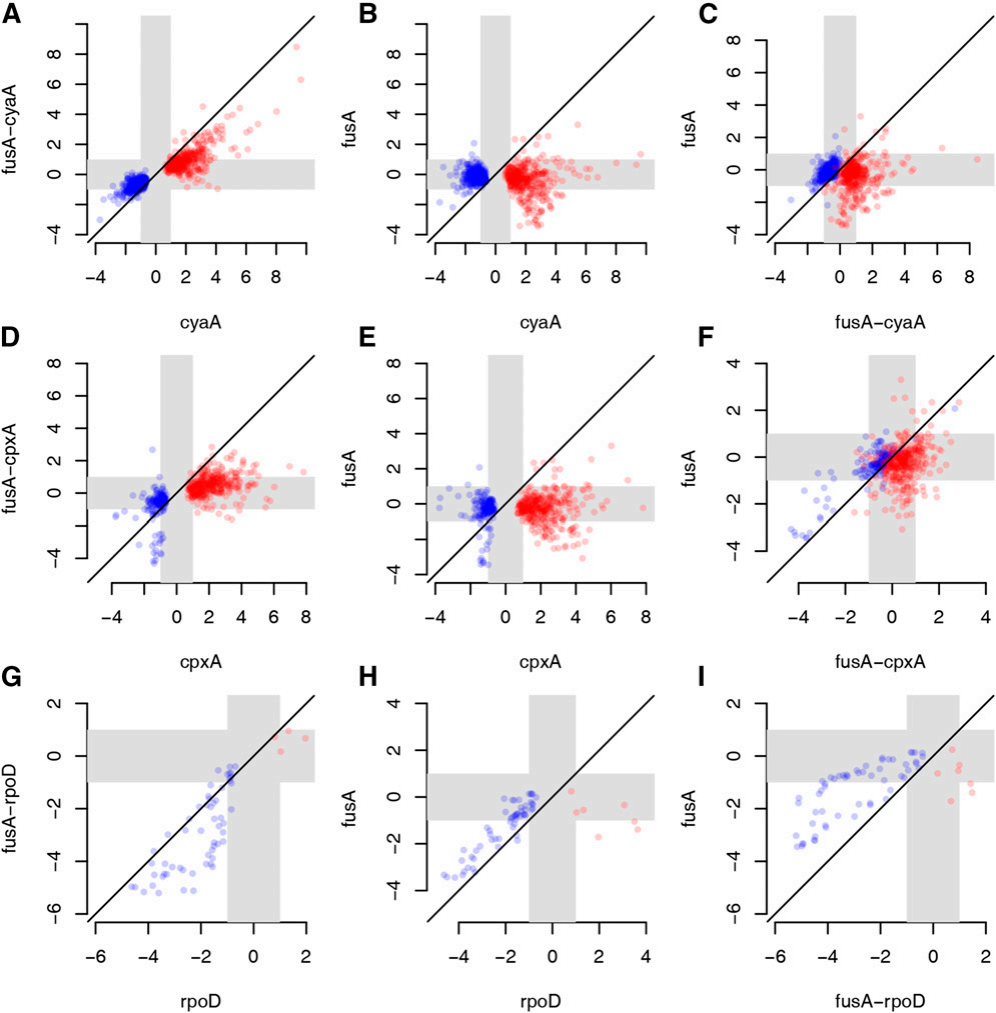
Effect of second site mutations on gene expression and dependence on EF-G. Scatter plots comparing log_2_ fold changes of differentially expressed genes among mutants. Mutants are referred to by their gene names for brevity. Gray zones indicate the region between the log_2_ fold changes of +1 and −1 (corresponding to fold changes of 2 and 0.5), and thus highlights the region of low/no fold change. Red points show genes upregulated and blue points show genes downregulated in the relevant second site single mutant. (A–C) Fold changes of genes differentially expressed in the CyaA^N600Y^ mutant were compared with the FusA^A608E^-CyaA^N600Y^ and FusA^A608E^ mutants. (D–F) Fold changes of genes differentially expressed in the CpxA^F218Y^ mutant were compared with the FusA^A608E^-CpxA^F218Y^ and FusA^A608E^ mutants. (G–I) Fold changes of genes differentially expressed in the RpoD^L261Q^ mutant were compared with the FusA^A608E^-RpoD^L261Q^ and FusA^A608E^ mutants.

More specifically, we saw that the fold changes of genes differentially expressed in the CyaA^N600Y^ mutant were reduced in the FusA^A608E^-CyaA^N600Y^ mutant (Figure 6A). Most of these genes were not differentially expressed in the FusA^A608E^ mutant (Figure 6, B and C). We saw that this was the case with the CpxA^F218Y^ and FusA^A608E^-CpxA^F218Y^ mutants as well (Figure 6, D–F). As a result of this, the number of differentially expressed genes in the FusA^A608E^-CyaA^N600Y^ or FusA^A608E^-CpxA^F218Y^ mutants is lesser than that in the CyaA^N600Y^ or CpxA^F218Y^ mutants (Figure 5A). The effect of the mutation in *fusA* on the fold changes of genes seems to be more extreme in the case of the mutation in *cpxA* than *cyaA*, since FusA^A608E^-CpxA^F218Y^ does not correlate as well with CpxA^F218Y^ as does FusA^A608E^-CyaA^N600Y^ with CyaA^N600Y^ (Figure 5B).

These observations stem, in part, from the fact that the fold changes of genes differentially expressed in the CyaA^N600Y^ and CpxA^F218Y^ mutants are opposite to the fold changes of these genes in the FusA^A608E^ mutant (Figure S16, A and B in File S2), even though they were not classified as differentially expressed in FusA^A608E^. In other words, a gene that is upregulated in CyaA^N600Y^/CpxA^F218Y^ displays a mild negative fold change in FusA^A608E^; whereas one that is downregulated in the former shows a slight positive fold change in the latter (Figure S16, A and B in File S2).

We notice that the effect of FusA^A608E^ on RpoD^L261Q^ is opposite to that on CyaA^N600Y^/CpxA^F218Y^. Genes downregulated in the RpoD^L261Q^ mutant were further downregulated in the FusA^A608E^-RpoD^L261Q^ mutant (Figure 6, G–I). In this case, many downregulated genes in RpoD^L261Q^ or FusA^A608E^-RpoD^L261Q^ were also downregulated in the FusA^A608E^ mutant (Figure 6, H and I and Figure S16C in File S2).

Unfortunately, we do not have information of this sort for the FusA^A608E^-TopA^S180L^ mutant since we did not have the corresponding second site single mutant.

Thus, the mutation in a translation elongation factor has a large effect on the transcriptional state of the cell, beyond that indicated by threshold-dependent calls of differential expression, presumably through feedback from levels of partially folded proteins.

### Genes with known roles to play in aminoglycoside resistance are misregulated in the mutants

Are the mechanisms of kanamycin resistance different or common across our set of mutants? To understand this, we looked at the kinds of genes differentially expressed in these mutants using GO or known transcription factor-target interactions to guide us.

Common trends in terms of shared gene functions are outlined in Figure 5C. In general, we found several metabolism related genes misregulated in the mutants. Misregulated genes with known roles in aminoglycoside resistance include genes involved in oxidative phosphorylation, protein folding and motility.

Genes involved in oxidative phosphorylation are downregulated in the FusA^A608E^ mutant. Oxidative phosphorylation produces reactive oxygen species (ROS), as a byproduct, which is thought to be involved in antibiotic mediated killing (Kohanski *et al.* 2007). A functional proton motive force generated by oxidative phosphorylation is required for aminoglycoside uptake (Taber *et al.* 1987). Furthermore, the components of the electron transport chain (ETC) tend to be Fe-S proteins, and are membrane associated. Mistranslation of membrane associated proteins induced by aminoglycosides, and, hence, their misfolding could affect the integrity of the cell membrane and result in hydroxyl radical mediated cell death (Kohanski *et al.* 2008). Misfolded versions of these proteins could also release Fenton reactive Fe^2+^, which, in turn, could again result in hydroxyl radical generation (Kohanski *et al.* 2007). Thus, there are many ways in which downregulating genes involved in oxidative phosphorylation, as in the FusA^A608E^ mutant, can alleviate the lethal effects of kanamycin.

Apart from these genes, genes associated with enterobactin biosynthesis or iron homeostasis are known to affect intracellular ROS levels (Méhi *et al.* 2014). It is possible that the upregulation of these genes in the FusA^A608E^, FusA^A608E^-CpxA^F218Y^, and FusA^A608E^-TopA^S180L^ mutants could reduce the production of ROS in the cells via sequestration of free Fe^2+^, and, hence, contribute to resistance.

The downregulation of oxidative phosphorylation and the upregulation of siderophore metabolism genes are in line with the hypothesis of oxidative damage mediated cell death in the presence of antibiotics (Kohanski *et al.* 2007). While this theory is fiercely disputed (Keren *et al.* 2013; Liu and Imlay 2013), it is possible that ROS aggravate the more direct effect of antibiotics, if not dominate it. For example, Ling *et al.* (2012) show that aminoglycoside induced protein aggregation is prevented by hydrogen peroxide quenchers. Dealing with ROS might result just in that extra protection that cells need in the presence of aminoglycosides.

ROS is a double edged sword. While it can damage cellular macromolecules and confer stress, the outcome of this stress could also result in an increase in mutagenesis (Kohanski *et al.* 2010). Thus, if these mutants do indeed reduce oxidative stress, it is quite possible that they will slow down further adaptation to kanamycin by reducing the occurrence of resistance conferring mutations; however, this remains to be tested.

We see a strong and consistent downregulation of motility associated genes, in all except the *cyaA* mutants. Using a transposon mutagenesis screen Shan *et al.* (2015), show that the loss of these genes results in decreased persister formation in aminoglycosides, and thus their downregulation in the mutants is contrary to our expectation. However these genes are upregulated in the CyaA^N600Y^ and FusA^A608E^-CyaA^N600Y^ mutants, and there they could contribute to resistance. Notably, motility genes are not found in the list of knockouts sensitive to aminoglycosides provided by Tamae *et al.* (2008), or in the list of loci that significantly affect susceptibility to aminoglycosides in the transposon insertion screen performed by Girgis *et al.* (2009).

Resistance to aminoglycosides can be provided by genes involved in protein transport/folding/secretion as these could help refold misfolded proteins (Goltermann *et al.* 2013). However, we see a downregulation of these genes in the FusA^A608E^-RpoD^L261Q^, FusA^A608E^-CpxA^F218Y^, and FusA^A608E^-TopA^S180L^ mutants, and this is contrary to our expectation.

### Conclusions

We saw that mutations that modify global transcriptional regulatory networks increase the resistance of the kanamycin-resistance conferring mutation in the translational elongation factor EF-G. These “second-site” mutations resulted in large changes in gene expression and displayed epistatic interactions with the mutation in EF-G, which itself drove expression changes of many genes. We show that these second site mutations reduce the activities of CyaA (adenylate cyclase) and TopA (topoisomerase I), and increase the activity of CpxA. Further evolution of an EF-G mutant in higher concentration of kanamycin suggested CpxA as the next target for an increase in resistance, with many high frequency mutations located in the helix-I domain of this protein. Although the activity of the mutated adenylate cyclase is reduced in the CyaA mutant that we isolated, many CRP targets are unexpectedly upregulated and this contradictory behavior needs further investigation. We suggest that FusA^A608E^-TopA^S180L^ results in a reduction in the function of topoisomerase I. Many genes with known roles in aminoglycoside resistance, for example, genes involved in oxidative phosphorylation and enterobactin metabolism, were misregulated in these mutants, thus pointing to possible mechanisms of resistance.

## Supplementary Material

Supplemental material is available online at www.g3journal.org/lookup/suppl/doi:10.1534/g3.117.300284/-/DC1.
