# Supplementary material for: Modulation of Global Transcriptional Regulatory Networks as a Strategy for Increasing Kanamycin Resistance of the Translational Elongation Factor-G Mutants in Escherichia coli
Source: G3 (Bethesda). 2017 Oct 18;7(12):3955–66. doi: 10.1534/g3.117.300284 (PMC5714492; doi:10.1534/g3.117.300284)
Supplement: Supplementary file 2 [file 3955FileS2.pdf]

Modulation of global transcriptional regulatory networks as a strategy for increasing kanamycin resistance of the translational Elongation Factor-G mutants

Aalap Mogre[1], Reshma T. V.[1, 2] and Aswin Sai Narain Seshasayee[1]

[1] National Centre for Biological Sciences, Tata Institute of Fundamental Research, GKVK, Bellary Road, Bangalore, Karnataka 560065, India

[2] The Institute of Trans-disciplinary Health Sciences and Technology, TDU, India

Corresponding Authors:

Aswin Sai Narain Seshasayee

National Centre for Biological Sciences, Tata Institute of Fundamental Research, GKVK, Bellary Road, Bangalore, Karnataka 560065, India

Email id: aswin@ncbs.res.in

Aalap Mogre

National Centre for Biological Sciences, Tata Institute of Fundamental Research, GKVK, Bellary Road, Bangalore, Karnataka 560065, India

Present address: Department of Microbiology, Moyne Institute of Preventive Medicine, School of Genetics and Microbiology, Trinity College Dublin, Dublin 2, Ireland.

Email id: aalapbm@ncbs.res.in

Supplementary Methods

Identification of SNPs from deep-sequencing data

The following pipeline was used to identify SNPs from genomic-DNA / RNA deep sequencing data. Reads were trimmed using CUTADAPT (Martin 2011) to remove adapter sequences. Trimmed reads with less than 30 bases were discarded. The remaining trimmed reads were aligned to the reference genome (NC_000913.3) using BWA (Li and Durbin 2010) with the -q 30 flag that trims bases from the ends of reads with qualities less than Phred score 30. SAMTOOLS version 1.3 (Li *et al.* 2009) was then used to generate the pileup file from the sam files generated by BWA. Finally, the list of single nucleotide polymorphisms (SNPs) and indels was compiled from the pileup file using VARSCAN version 2.3.8 (Koboldt *et al.* 2012). The list of mutations was filtered such that the mutations present in at least 90% of the reads, in at least one sample, were retained. Mutations present in the wildtype were filtered out.

In the RNA-seq data, ideally, the mutants should also have a mutation in *yhgA* as was shown in the genome sequencing done in our previous work (Mogre *et al.* 2014). However, the expression level of this gene is very low—only a few reads cover these genes. Since the mutated site is not covered this mutation is not called. We see the mutation in *yhgA* in the $FusA^{A608E}$-$TopA^{S180L}$ second replicate though. We also see varying levels of a mutation in *obgE* (non-synonymous resulting in the residue change S75T) in some of the strains. This apparently mutated locus had low coverage and was mostly supported by bases towards the ends of reads. We re-analyzed the data by trimming 10 bases from both ends of the reads. After doing this, the mutation in *obgE* disappeared from most strains (Fig. S2B). Thus we think that the mutation is an artifact of sequencing. We confirmed that the mutation is absent by Sanger sequencing (Fig. S2C).

Measuring variability in expression of genes within an operon

As an additional check of our RNA-seq data, we determined whether genes within operons were similarly expressed. We obtained the list of operons from the DOOR[2] database of prokaryotic operons (Mao *et al.* 2009). We normalized the gene read count to gene length and used this as a measure of expression of each gene. To measure the variation in expression of genes within operons, we calculated the population standard deviation (SD) of the expression values of genes within operons, individually for each operon. The formula for population standard deviation was used since a large number of operons contained only two genes (division by n instead of n-1). These operon specific measures of variation in gene expression were divided by the genome-wide variation in gene expression (i.e. the total SD across all genes for that sample, referred to as Total SD in the plots). If the within-operon SD is lower than the total SD, these values will be below 1. To check if this could arise by random chance, we randomly sampled genes equal to the number of genes in each operon. For all these sets of randomly sampled genes, we performed this calculation again. These values were also less than 1. This was because the total SD was very high, and that high variation was driven by few highly / lowly expressed genes. These genes tend to not be sampled during the random sampling because they are fewer in number. However, the median in the “Random” dataset, was much higher than that of the

"Real" dataset. The difference between the two was checked using the two-sided Wilcoxon rank sum test. In all samples, the P values for comparisons between the "Real" and "Random" dataset were below 1.0×10^{-55}.

Link for DOOR[2]: http://csbl.bmb.uga.edu/DOOR/displayNCoperon.php?id=1944&page=1&nc=NC_000913#tabs-1

Cell imaging and cell length estimation

Overnight grown bacterial cultures were diluted 1:100 in lysogeny broth (LB, with or without kanamycin) and grown in 96 well plates (100 µl per well) incubated at 37 °C, 200 rpm for 24 hours. Cells were pelleted by centrifugation at 4000 rpm for 3 minutes and resuspended in twice the volume of phosphate buffered saline (PBS, pH 7). These cells were then embedded under a 1% agarose pad prepared by dissolving agarose (Invitrogen) in PBS. The embedded cells were imaged using a NIKON eclipse Ti2 inverted microscope. Phase contrast images were taken using a 60× lens with oil immersion and analyzed using Oufti (Paintdakhi *et al.* 2016) with the *E.coli*_LB_subpixel.set parameters. The cell length in number of pixels was converted to microns by multiplying with a pixel to micron conversion factor (0.064). Around 725 cells of each strain were imaged from multiple experiments (n = 4) to obtain a distribution of cell lengths.

Spotting experiments to measure colony forming units (CFUs)

Overnight grown bacterial cultures were diluted 1:100 in lysogeny broth (LB, with or without kanamycin) and grown in 96 well plates (100 µl per well) incubated at 37 °C, 200 rpm for 24 hours. Serial 10-fold dilutions of these cultures in 0.75 % saline were spotted (as 5 µl spots) on LB agar plates. The plates were imaged after 14 hours of incubation at 37 °C.

Measurements of promoter activities

This assay utilizes a plasmid (pMS201) carrying a reporter gene (GFP^{mut2}) downstream of the cloned promoters of genes. These plasmids were extracted from the Thermo Scientific *E. coli* promoter collection (Zaslaver *et al.* 2009). These plasmids were then introduced in the wildtype (WT with FRT site near *fusA*) and $CyaA^{N600Y}$ mutant (which also has FRT site near *fusA*). In this assay, GFP^{mut2} expression, driven by the promoters of *cyaA* and *crp* report the activities of these promoters. Simultaneous measurement of optical density at 600 nm (OD_{600}) and fluorescence (excitation wavelength: 485 nm, emission wavelength: 520 nm) of the strains were performed in black 96-well plates, every 15 minutes, with the Tecan Infinite F200 pro plate reader, with shaking at 198 rpm and incubation at 37 ^{0}C. We saw that the activities of the promoters of *cyaA* and *crp* were higher in the mutant than the wildtype. Promoter activities were calculated as thus: Blank subtracted fluorescence values were divided by blank subtracted OD_{600} values. Similarly OD_{600} normalized fluorescence values from promoter-less strains were subtracted from these values (background subtraction, where background refers to promiscuous GFP expression from promoter-less plasmid). Promoter activity was then calculated as the temporal derivative of these fluorescence values (dF/dt). Subsequently, for each gene separately, the promoter activities in the WT and $CyaA^{N600Y}$ strains were scaled to fall between 0 and 1 before plotting.

Conservation analysis of RpoD

Phmmer (using the –notextw flag to facilitate sequence comparison) was used to find the best hit to the wildtype RpoD in *E. coli* K12 MG1655 in each of the 2074 bacterial genomes used in the analysis. Next, from each of these hits, for each residue, the number of times each residue in the hit matched the corresponding wildtype residue was counted and this count was divided by the total number of genomes analyzed to obtain a conservation score. Then we scaled the data such that it would fall between 0 and 1. This does not change the picture, only changes the scale.

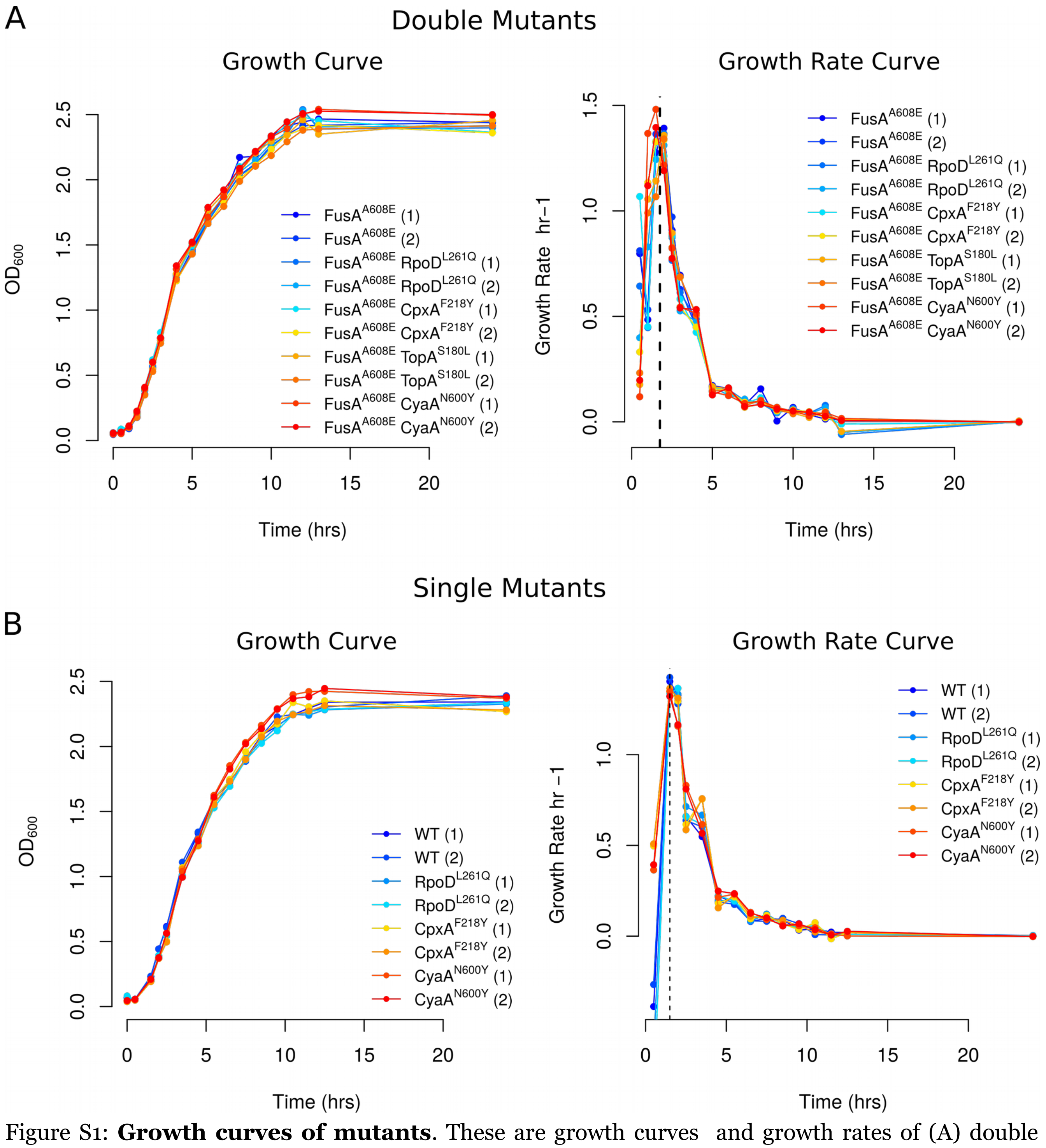

Figure S1: **Growth curves of mutants**. These are growth curves and growth rates of (A) double mutants and (B) single mutants, grown in LB. Replicate number is mentioned in parenthesis. The time-point at which cells were harvested for RNA extraction is indicated by a dashed line in the growth rate curves.

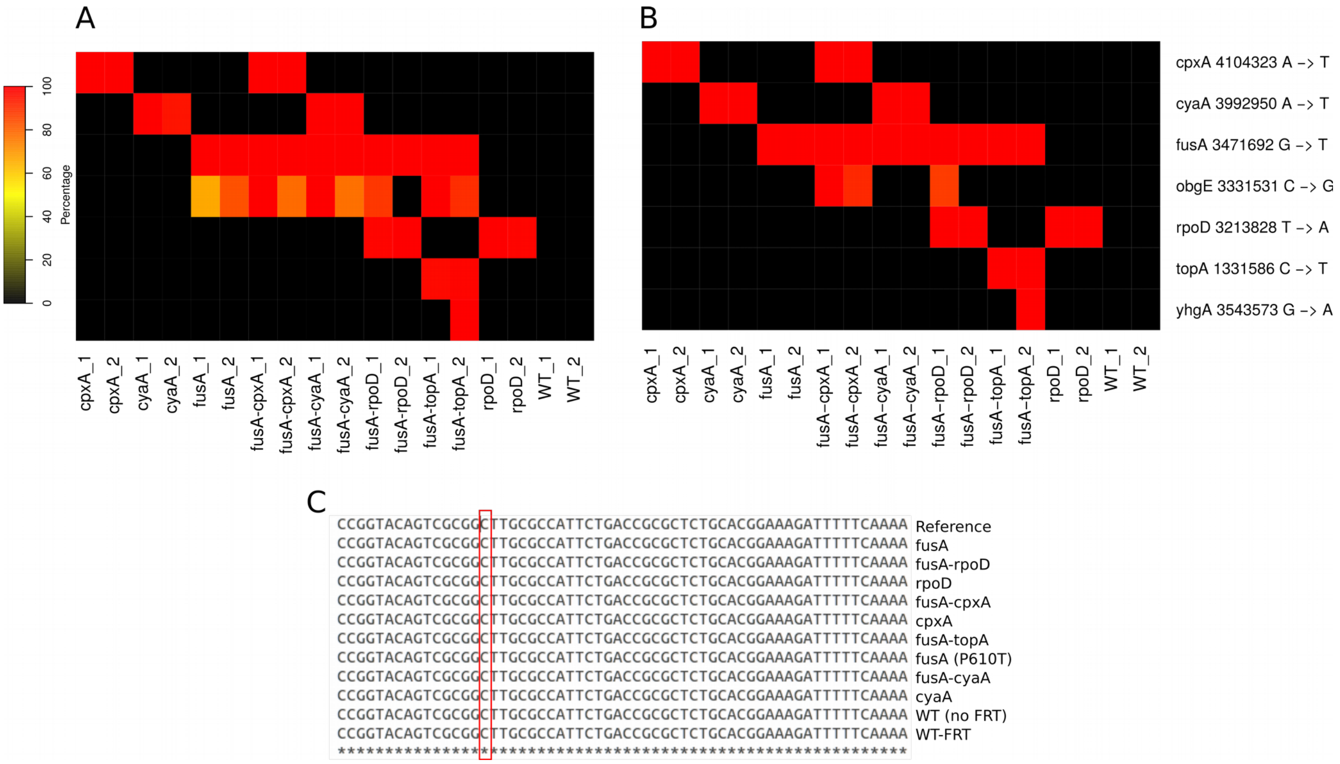

Figure S2: **SNPs called from RNA-seq data confirm identity of mutants.** (A) Heatmap showing the percentage of reads supporting mutations (SNP frequency) in the RNA-seq data of the mutants. Replicates are mentioned by numbers. (B) Heatmap showing the SNP frequency in RNA-seq data after trimming the read ends by 10 bases. (C) Sanger sequencing of the *obgE* SNP locus.

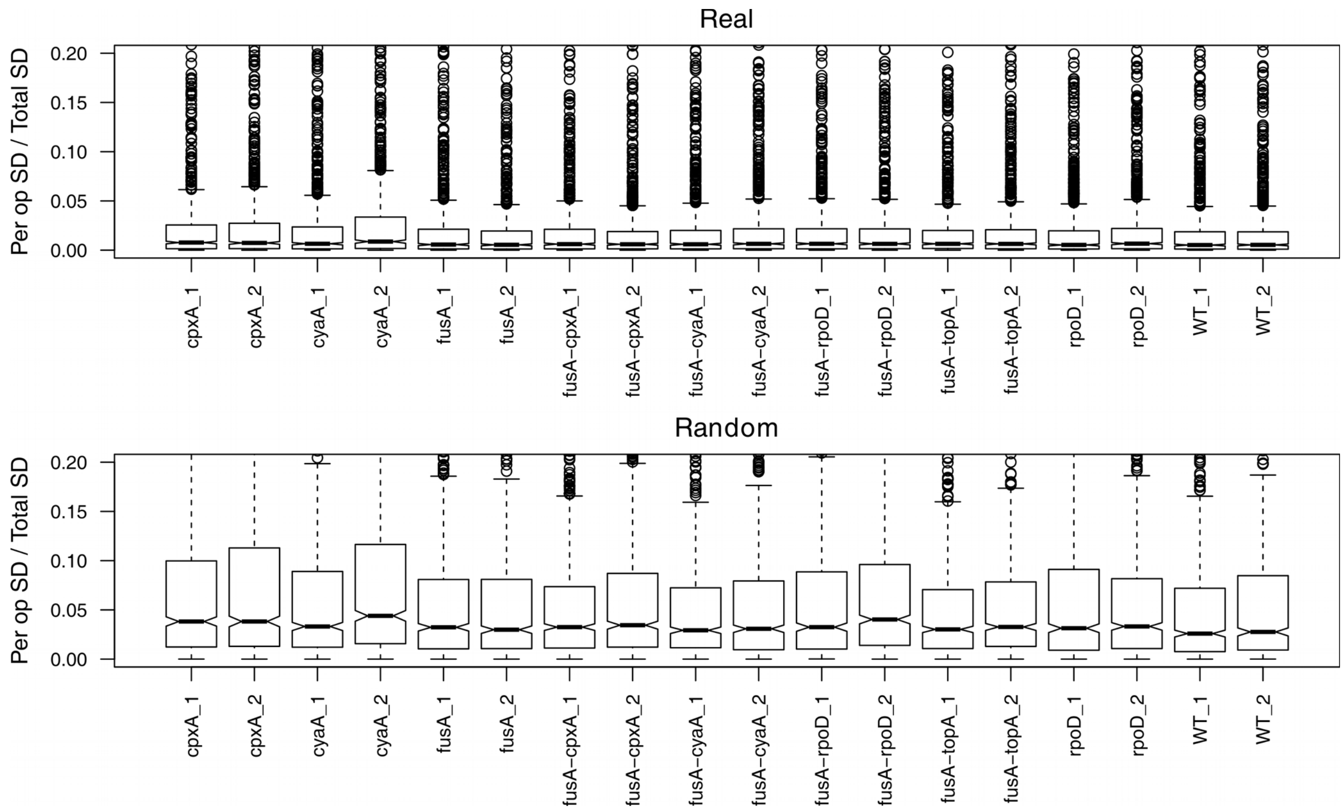

Figure S3: **Low variation in expression of genes within operons.** Boxplots representing the distributions of the ratios of within operon SD to total SD (see Supplementary Methods). In all samples the P values for comparisons between the “Real” and “Random” dataset were below 1.0 x 10^{-55} (two-sided Wilcoxon rank sum test).

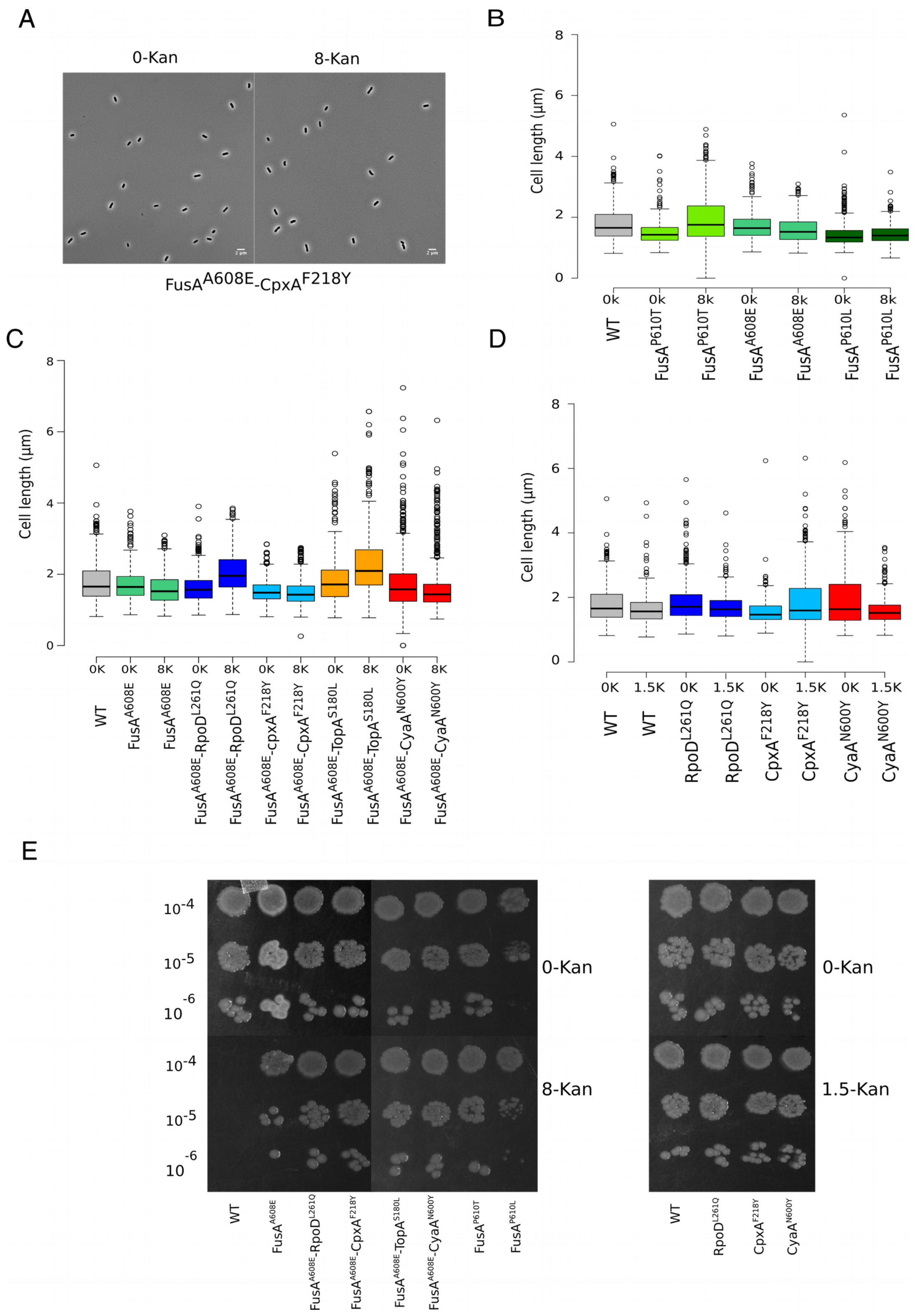

Figure S4: **Cell length and CFU measurements of strains.** (A) Representative 60× phase contrast microscopy images of a mutant (FusA^{A608E}-CpxA^{F218Y}) in the presence and absence of kanamycin. (B-D) Boxplots showing distributions of cell lengths of strains in the presence and absence of kanamycin. (E) Spotting assay for WT and various mutants to compare the growth in presence and absence of kanamycin. See Supplementary Methods for further details of the protocols involved in these experiments.

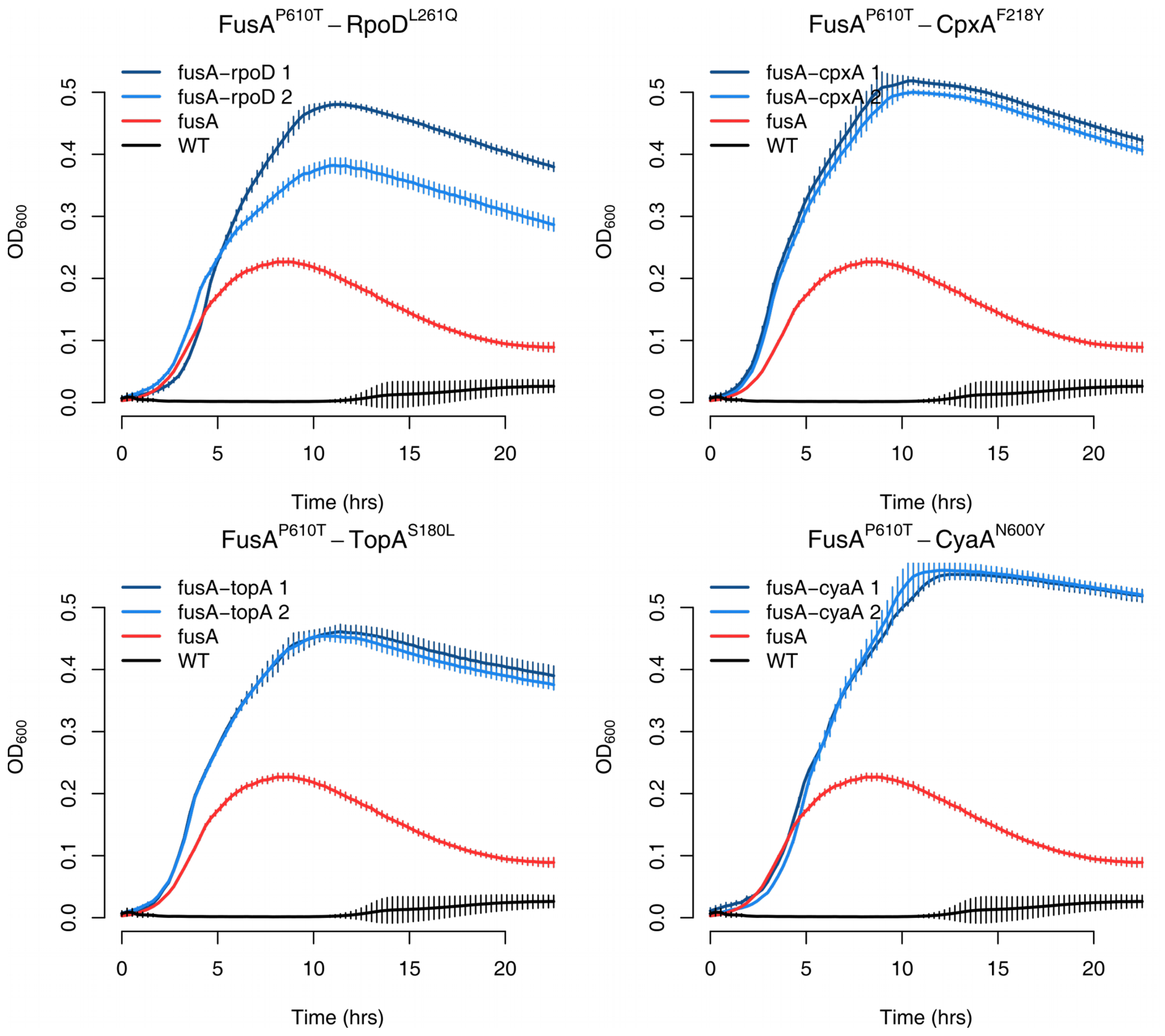

Figure S5: **Second site mutations, evolved in the FusA^{A608E} background, confer growth advantage in kanamycin in the FusA^{P610T} background as well.** Growth of FusA^{P610T}-RpoD^{L261Q} / CpxA^{F218Y} / TopA^{S180L} / CyaA^{N600Y} mutants in LB containing 8 μg/ml kanamycin (~50 % lethal concentration for the wildtype, 8-kan). Optical densities and standard deviation obtained from eight replicates is plotted. The second FusA^{P610T}-RpoD^{L261Q} transductant seems to have a slight growth defect which is seen in the absence of kanamycin as well (Fig. S6) and could be due to accumulation of some other mutation during the strain generation process.

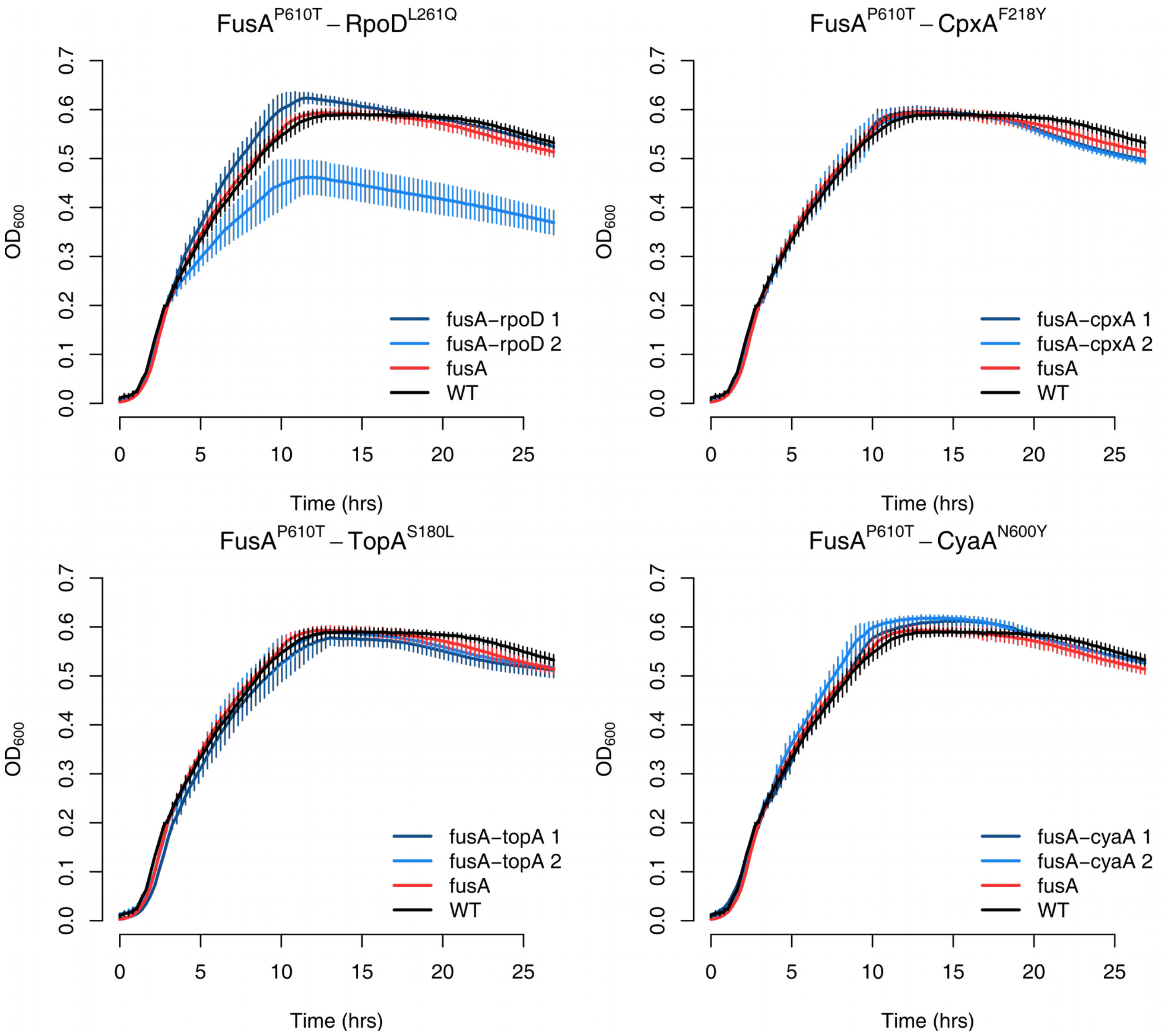

Figure S6: **Lack of growth defect in the $FusA^{P610T}$- $RpoD^{L261Q}$ / $CpxA^{F218Y}$ / $TopA^{S180L}$ / $CyaA^{N600Y}$ mutants.** Growth of $FusA^{P610T}$-$RpoD^{L261Q}$ / $CpxA^{F218Y}$ / $TopA^{S180L}$ / $CyaA^{N600Y}$ mutants in LB. Optical densities and standard deviations obtained from eight replicates are plotted.

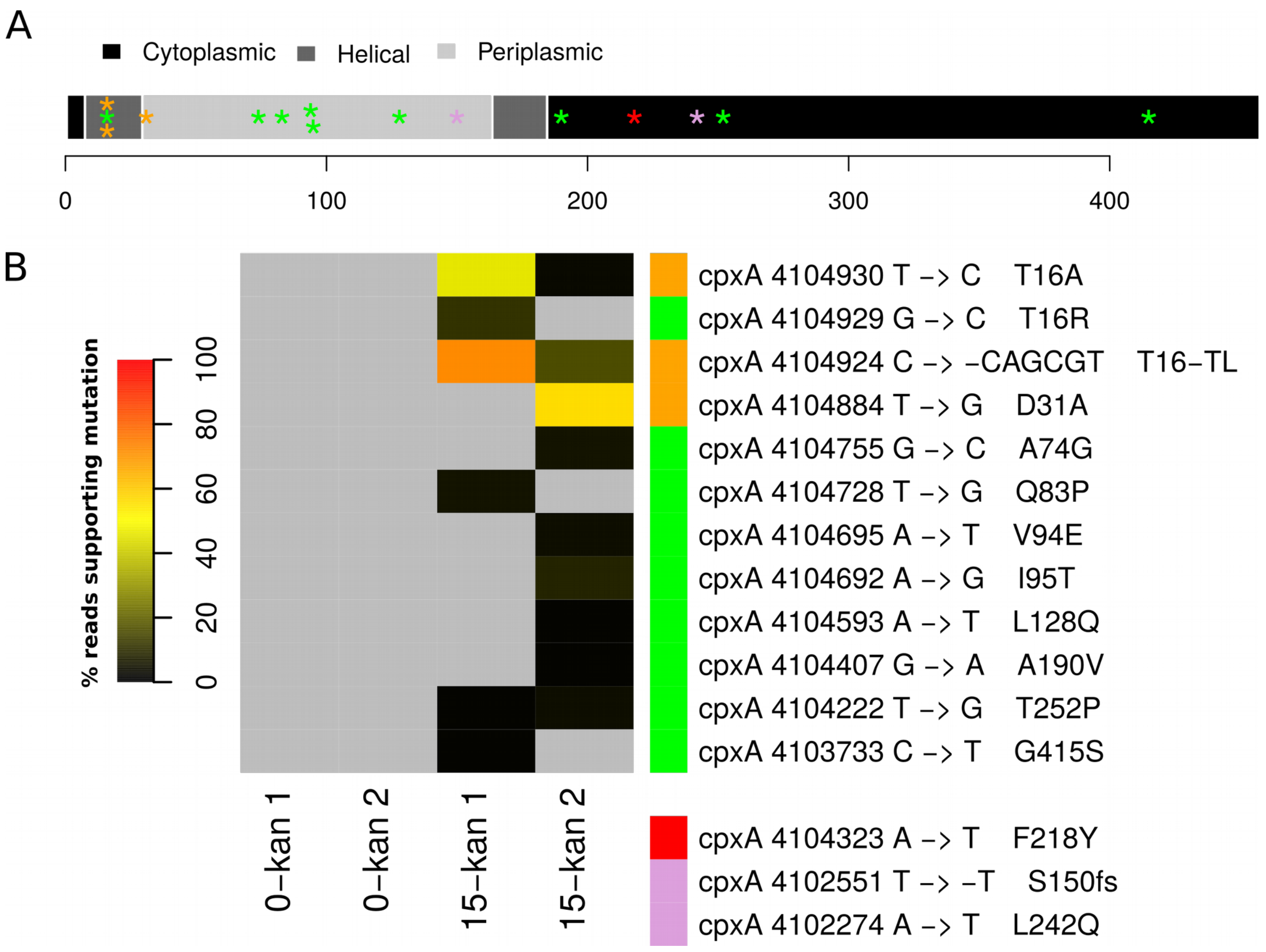

Figure S7: **Mutations in *cpxA* that increase in frequency above 50% in the FusA^{P610T} populations evolving in 15 µg/ml kanamycin (15-kan) are confined to the helix I region of CpxA.** (A) The domain structure of CpxA as obtained from Uniprot (http://www.uniprot.org/uniprot/P0AE82). The coloured "*"s mark the mutations in *cpxA* that confer kanamycin resistance.

Red "*" marks the mutation obtained in our previous work, and which we have studied in detail in this work.

Plum "*"s are mutations seen by Lazar et al. (Lázár *et al.* 2013).

Green and orange "*"s mark mutations obtained during the evolution of FusA^{P610T} populations in 15-kan, described in this work. The orange "*"s mark mutations that are present in greater than 50% of reads in at least one population, whereas the green "*"s mark mutations that are present in less than 11% of the reads.

(B) Heatmap showing the percentage of reads supporting mutations in *cpxA*. These mutations are also marked in the domain structure of CpxA in (A). The coloured bar on the right shows the colour code of the mutations marked in (A). (The position number of the Lazar et al. mutants are based on the NC_000913.2 reference genome.)

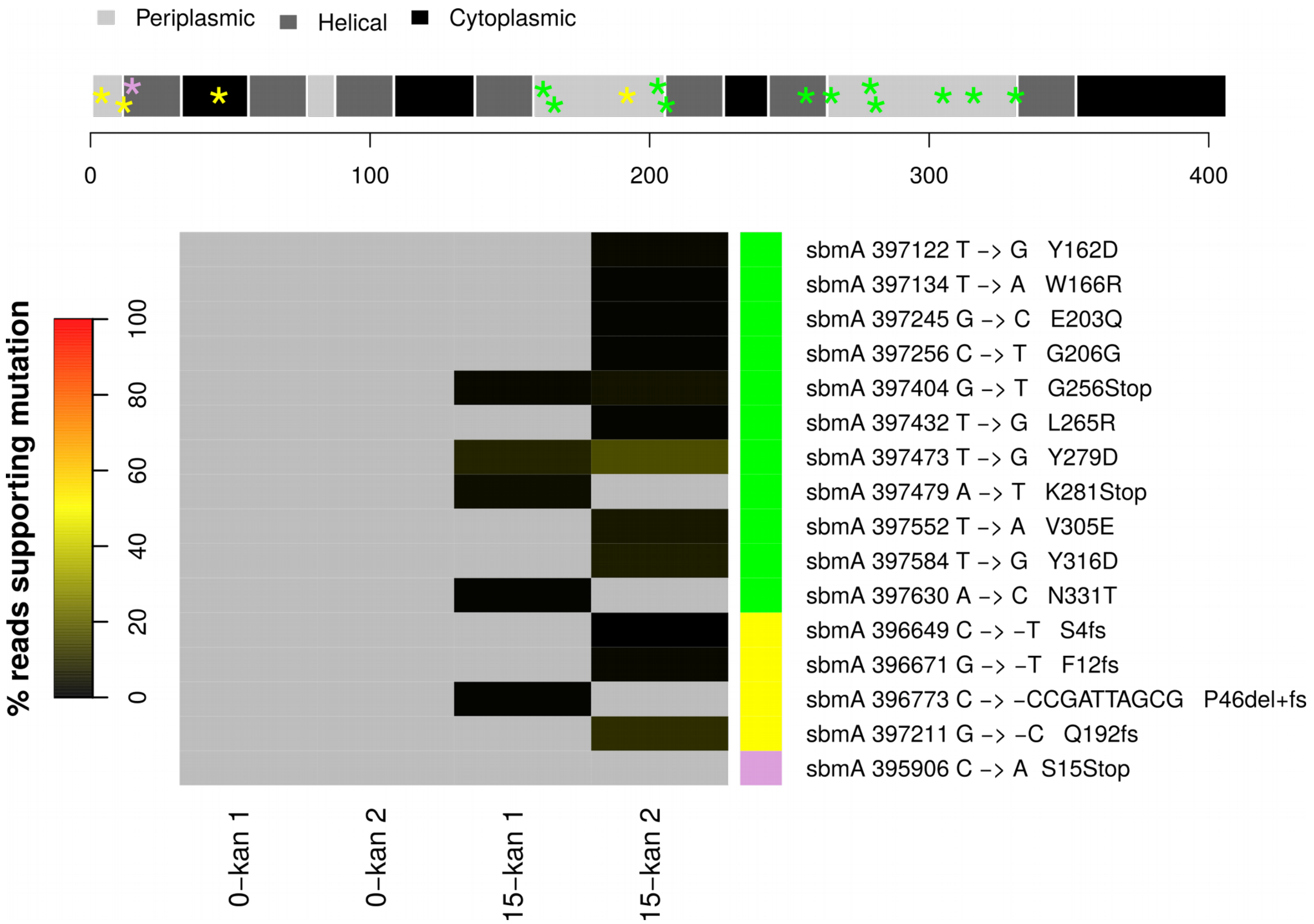

Figure S8: **Mutations in *sbmA* are present in low levels in the FusA^{P610T} populations evolved in 15 µg/ml kanamycin (15-kan).** (A) Domain structure of SbmA as obtained from Uniprot (http://www.uniprot.org/uniprot/P0AFY6) showing the location of mutated residues with "*"s.

Green "*"s are SNPs.
Yellow "*"s are deletions.

Plum "*" is the mutation in *sbmA* seen by Lazar *et al.* (Lázár *et al.* 2013).

(B) Heatmap showing the percentage of reads supporting various mutations in the gene *sbmA*. The coloured bar on the right shows the colour code of the mutations marked in (A). (The position number of the Lazar et al. mutants are based on the NC_000913.2 reference genome.)

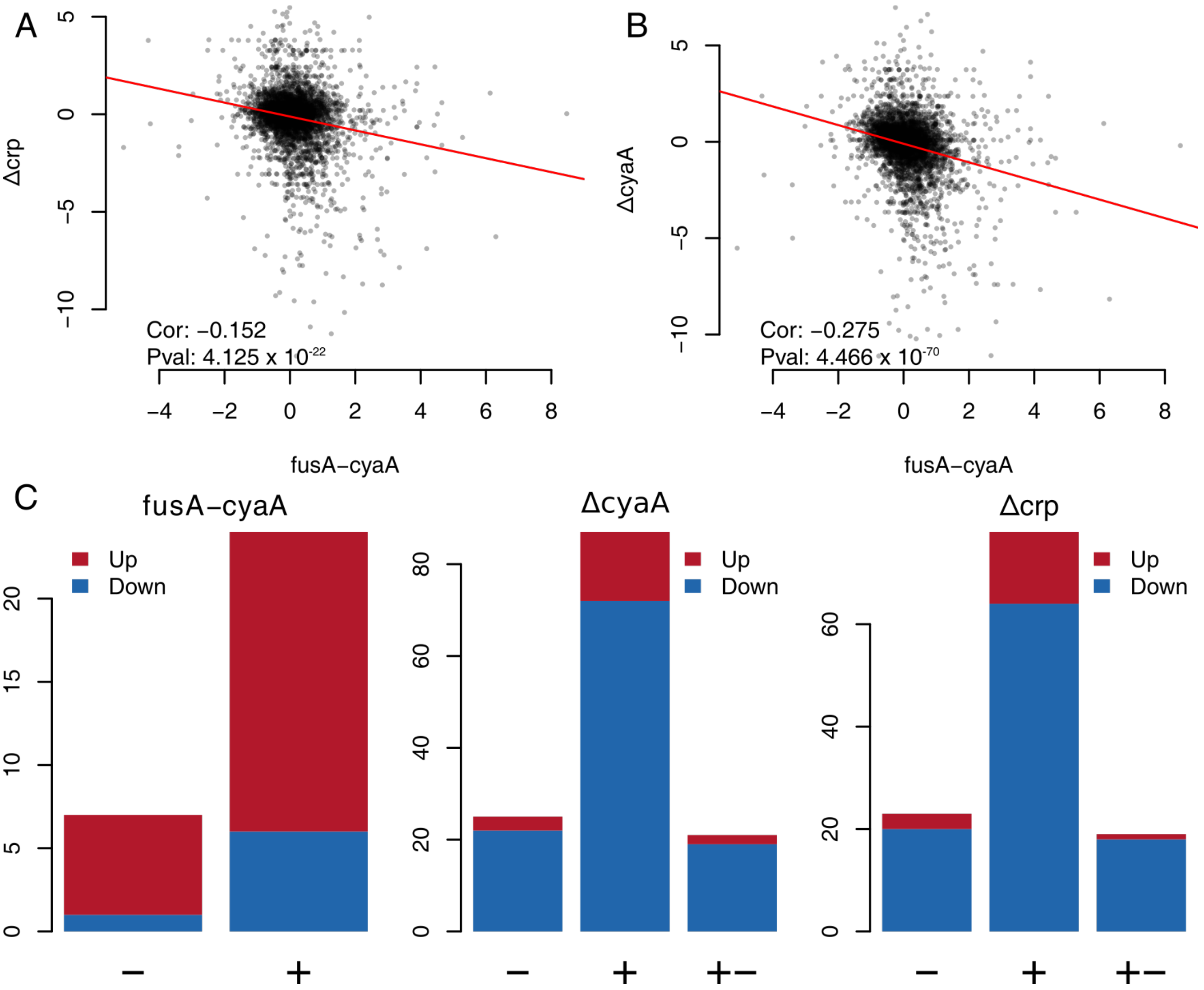

Figure S9: **Slight induction of the CRP regulon in the FusA^{A608E}-CyaA^{N600Y} mutant.** (A-B) Scatter plots comparing Log_2 fold-changes of genes in the FusA^{A608E}-CyaA^{N600Y} (fusA-cyaA) mutant with those in the Δ*crp* (A) / Δ*cyaA* (B) knockout strains. Spearman correlation coefficients and their P values are mentioned in the plots. (C) The numbers of positive (+), negative (-) and dual targets (+-) of CRP present among the up (red) and down-regulated (blue) genes of strains.

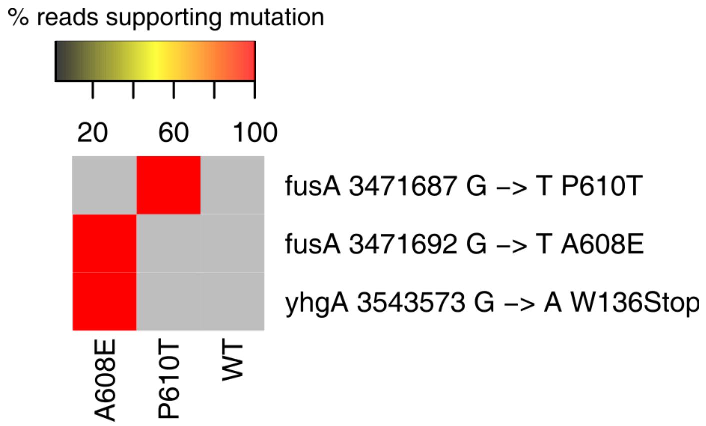

Figure S10: **SNP analysis of Δ*cyaA* strains.** Heatmap showing the percentage of reads supporting mutations in the WT-Δ*cyaA*, $FusA^{P610T}$-Δ*cyaA*, and $FusA^{A608E}$-Δ*cyaA* strains. The deletion of *cyaA* in all the three strains was confirmed by looking at the read coverage of the *cyaA* gene and is not shown here.

CyaA^{N600Y}	Log_2FC	P value
cyaA	0.564	0.018
crp	0.937	0.001

FusA^{A608E}-CyaA^{N600Y}	Log_2FC	P value
cyaA	0.591	0.029
crp	0.845	0.009

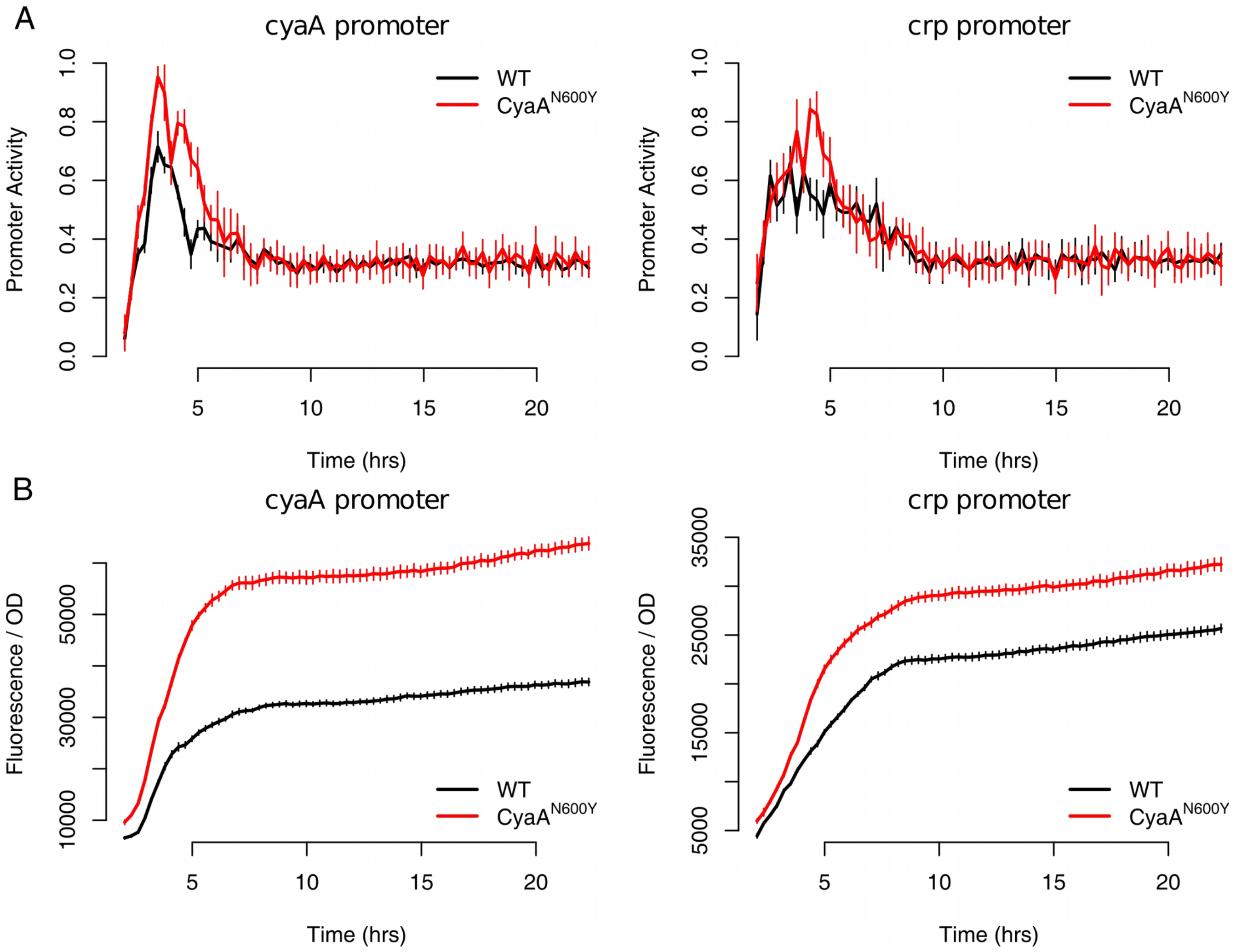

Figure S11: **Increased expression of *cyaA* and *crp* in the CyaA^{N600Y} mutant.** The two tables show the log_2 fold changes of the genes *cyaA* and *crp* in the CyaA^{N600Y} and FusA^{A608E}-CyaA^{N600Y} strains. (A) Promoter activities of *cyaA* and *crp* promoters in the wildtype (WT) and the CyaA^{N600Y} mutant. (B) GFP^{mut2} production (driven by above mentioned promoters) in the same. Error bars represent standard deviation.

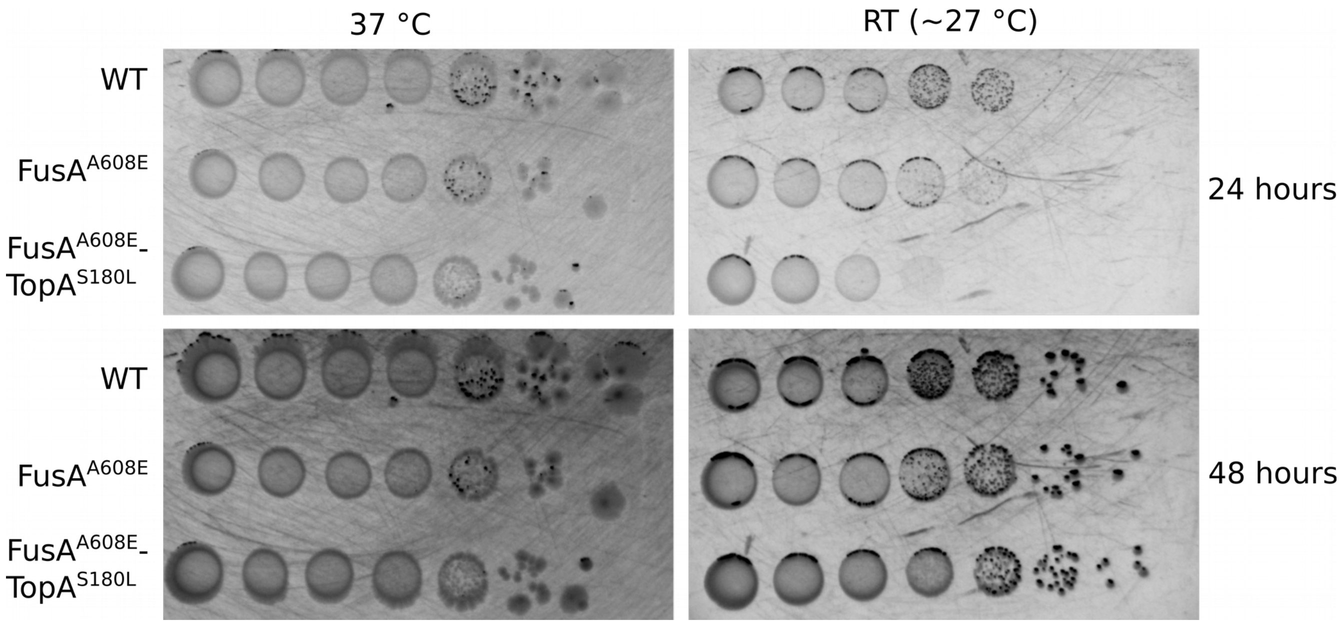

Figure S12: **Cold sensitivity of the FusA^{A608E}-TopA^{S180L} mutant.** Growth of 10-fold dilutions of overnight cultures on LB agar incubated at 37 °C or room temperature (RT), after 24 and 48 hours of incubation.

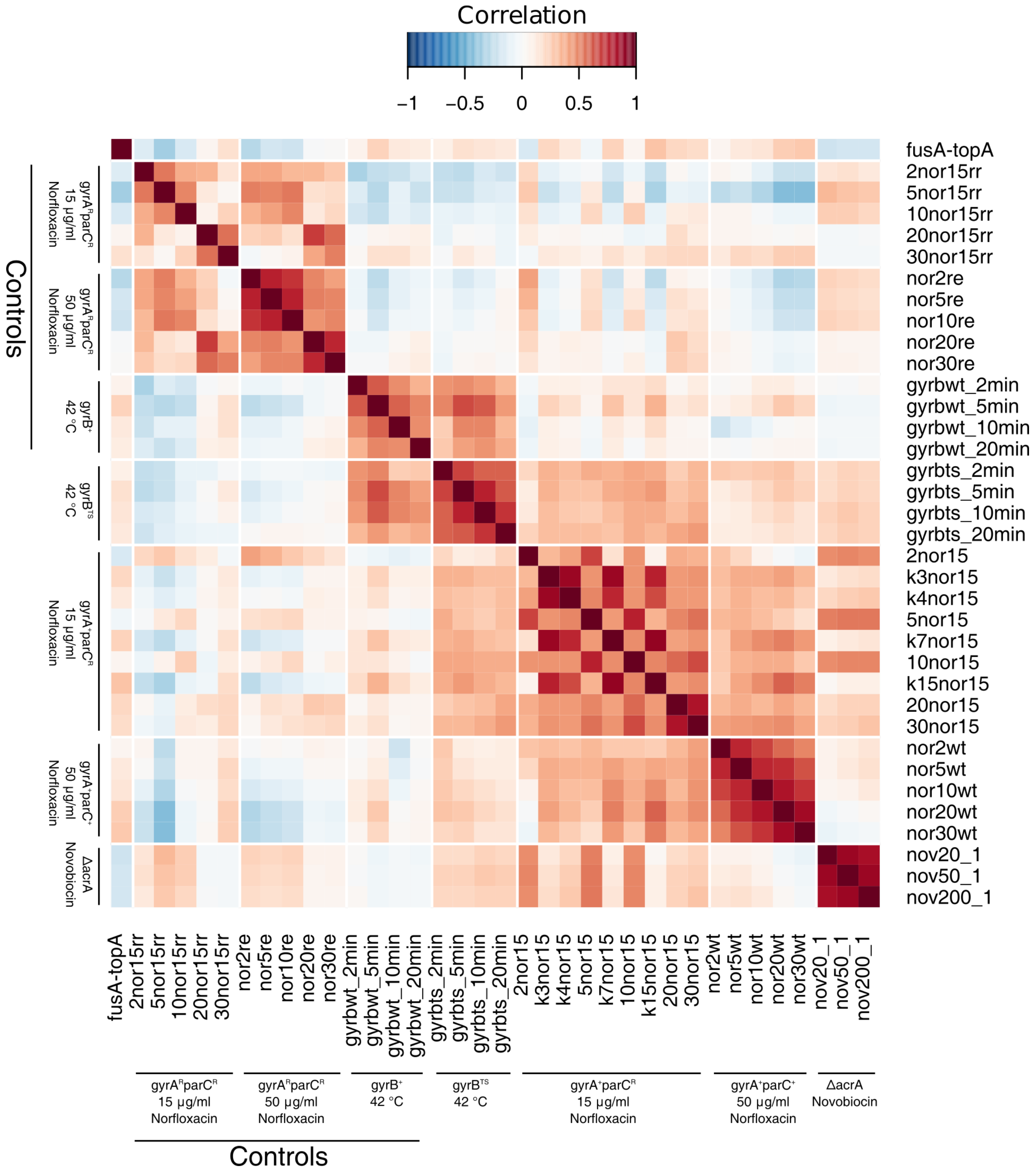

Figure S13: **Correlations with the Peter et al. Dataset.** Heatmap showing the strength of correlations of the transcription profile of the FusA^{A608E}-TopA^{S180L} mutant (fusA-topA) with those of *E. coli* strains grown under different conditions that result in reduced negative supercoiling (Peter *et al.* 2004).

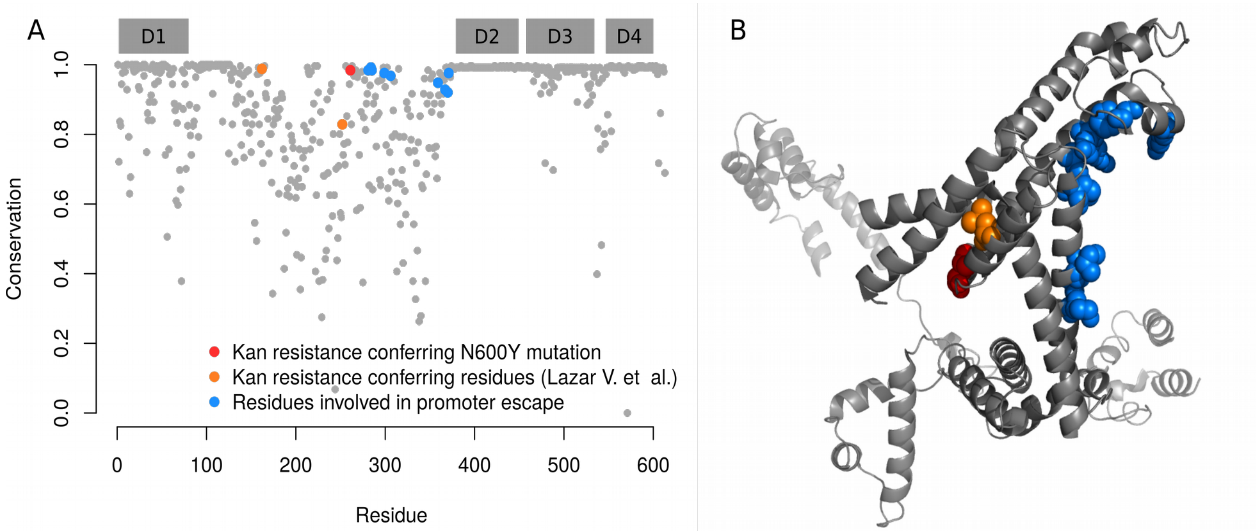

Figure S14: **Kanamycin resistance conferring mutations are located in well conserved residues in the non-conserved region of RpoD.** (A) This plot shows the conservation of each residue in RpoD. The kanamycin resistance conferring mutation in red was obtained in our previous study and is analysed further in this study. The residues shown in orange are kanamycin resistance conferring mutations found by Lazar et al (Lázár *et al.* 2013). The residues shown in blue have been identified in aiding promoter escape of the RNA polymerase (Leibman and Hochschild 2007). (B) Mutated residues, and residues involved in promoter escape, are shown on the crystal structure of RpoD (PDB structure 4KMU). Colour codes of residues match that of (A). The L261Q mutation seen by us is shown in red. The L252Q mutation seen by Lazar et al. is shown in orange. The I162N mutation, seen by Lazar et al., could not be shown as the residue was absent in the structure.

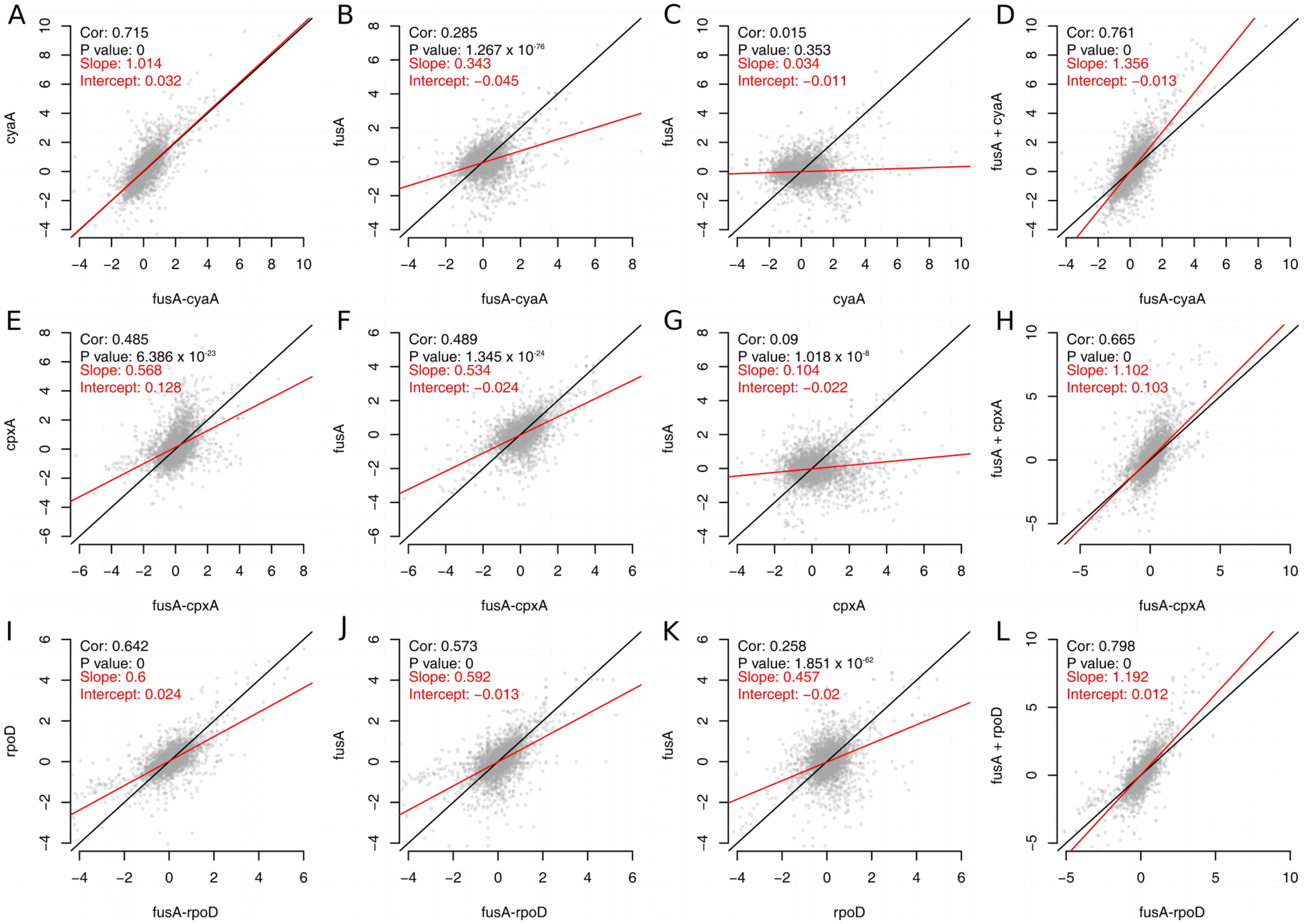

Figure S15: **Effect of second site mutations on gene expression and dependence on EF-G.** These are scatter plots comparing fold changes of genes among mutants. Unlike in Fig. 6, all genes are included in these plots. Mutants are referred to by their gene names for brevity. The 45° line is shown in black whereas the linear fit is shown in red. Values of Spearman correlation, their significance, slope and intercept of the linear fit are mentioned in each plot. (A-C) Correlations between FusA^{A608E}-CyaA^{N600Y}, CyaA^{N600Y} and FusA^{A608E} mutants. (D) Correlation between the FusA^{A608E}-CyaA^{N600Y} mutant and the addition of fold changes of genes in the FusA^{A608E} and CyaA^{N600Y} mutants. (E-G) Correlations between FusA^{A608E}-CpxA^{F218Y}, CpxA^{F218Y} and FusA^{A608E} mutants. (H) Correlation between the FusA^{A608E}-CpxA^{F218Y} mutant and the addition of fold changes of genes in the FusA^{A608E} and CpxA^{F218Y} mutants. (I-K) Correlations between FusA^{A608E}-RpoD^{L261Q}, RpoD^{L261Q} and FusA^{A608E} mutants. (L) Correlation between the FusA^{A608E}-RpoD^{L261Q} mutant and the addition of fold changes of genes in the FusA^{A608E} and RpoD^{L261Q} mutants. In all cases the absolute of the difference of, the sum of the $\log_2$ fold changes of genes in the FusA^{A608E} and CyaA^{N600Y}/ CpxA^{F218Y} / RpoD^{L261Q} single mutants, with the $\log_2$ fold changes of FusA^{A608E}-CyaA^{N600Y}/ CpxA^{F218Y} / RpoD^{L261Q} double mutants is significantly greater than zero (Wilcoxon signed rank test P value $< 2.2 * 10^{-16}$).

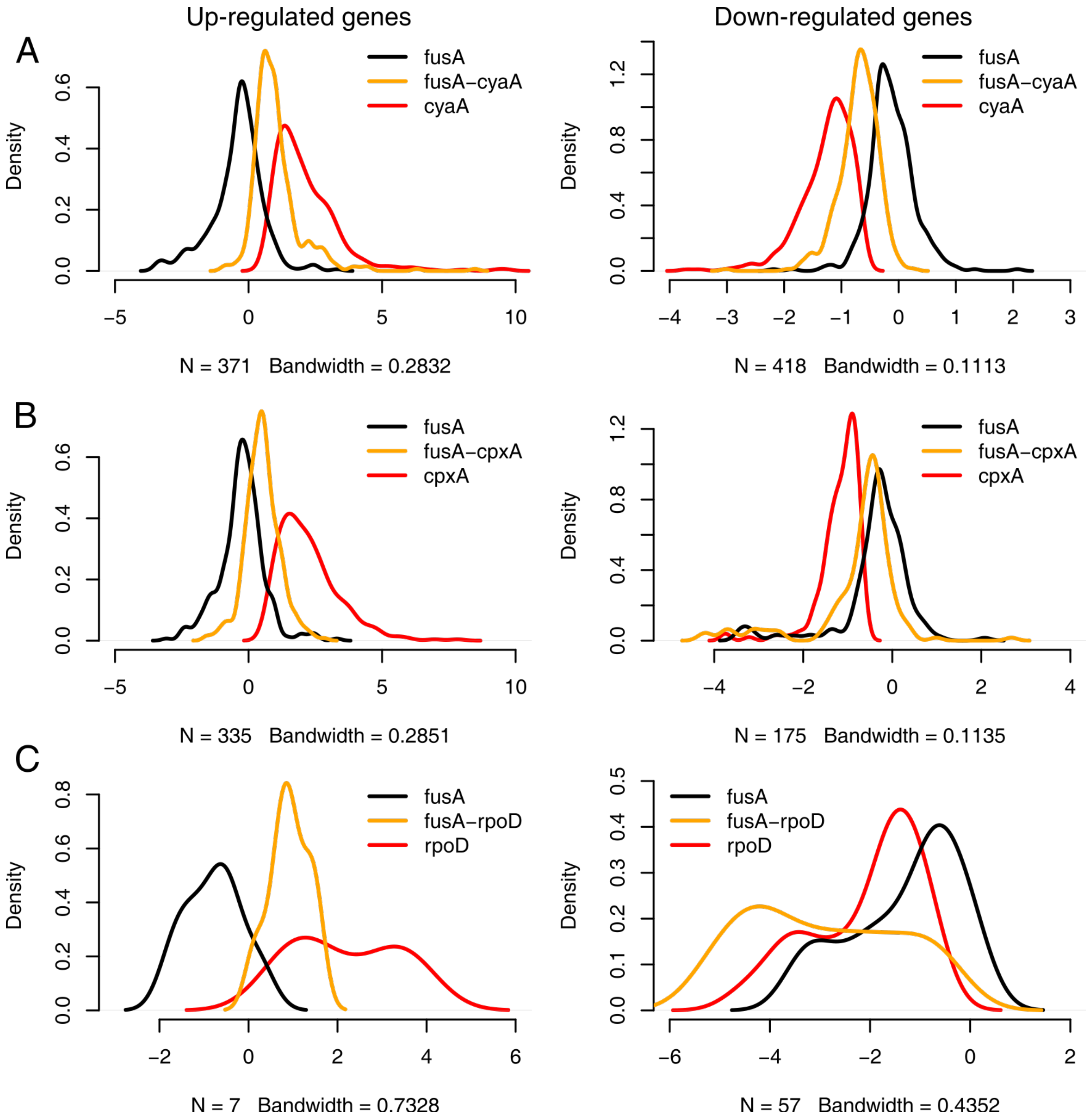

Figure S16: **Fold changes of genes, differentially expressed in the second site mutants, in the FusA^{608E} single and double mutants.** Kernel density plots of $\log_2$ fold changes of either up (left panel) or down-regulated (right panel) genes in the CyaA^{N600Y} (A), CpxA^{F218Y} (B) and RpoD^{L261Q} (C) mutants (all in red). Kernel density plots of $\log_2$ fold changes for these genes in the FusA^{A608E} single mutant (black) and FusA^{A608E}-CyaA^{N600Y} (A) / CpxA^{F218Y} (B) / RpoD^{L261Q} (C) double mutants (all in orange) are also shown.

References

Koboldt D. C., Zhang Q., Larson D. E., Shen D., McLellan M. D., *et al.*, 2012 VarScan 2: somatic mutation and copy number alteration discovery in cancer by exome sequencing. Genome Res. 22: 568–576.

Lázár V., Pal Singh G., Spohn R., Nagy I., Horváth B., *et al.*, 2013 Bacterial evolution of antibiotic hypersensitivity. Mol. Syst. Biol. 9: 700.

Leibman M., Hochschild A., 2007 A sigma-core interaction of the RNA polymerase holoenzyme that enhances promoter escape. EMBO J. 26: 1579–1590.

Li H., Handsaker B., Wysoker A., Fennell T., Ruan J., *et al.*, 2009 The Sequence Alignment/Map format and SAMtools. Bioinforma. Oxf. Engl. 25: 2078–2079.

Li H., Durbin R., 2010 Fast and accurate long-read alignment with Burrows-Wheeler transform. Bioinforma. Oxf. Engl. 26: 589–595.

Mao F., Dam P., Chou J., Olman V., Xu Y., 2009 DOOR: a database for prokaryotic operons. Nucleic Acids Res. 37: D459-463.

Martin M., 2011 Cutadapt removes adapter sequences from high-throughput sequencing reads. EMBnet.journal 17: 10–12.

Mogre A., Sengupta T., Veetil R. T., Ravi P., Seshasayee A. S. N., 2014 Genomic analysis reveals distinct concentration-dependent evolutionary trajectories for antibiotic resistance in Escherichia coli. DNA Res. Int. J. Rapid Publ. Rep. Genes Genomes 21: 711–726.

Paintdakhi A., Parry B., Campos M., Irnov I., Elf J., *et al.*, 2016 Oufti: an integrated software package for high-accuracy, high-throughput quantitative microscopy analysis. Mol. Microbiol. 99: 767–777.

Peter B. J., Arsuaga J., Breier A. M., Khodursky A. B., Brown P. O., *et al.*, 2004 Genomic transcriptional response to loss of chromosomal supercoiling in Escherichia coli. Genome Biol. 5: R87.

Zaslaver A., Kaplan S., Bren A., Jinich A., Mayo A., *et al.*, 2009 Invariant distribution of promoter activities in Escherichia coli. PLoS Comput. Biol. 5: e1000545.
